# Green synthesis of silver nanoparticles using *Solanum lycopersicum* leaves extract for highly selective detection of mercury ions and photocatalytic degradation of methylene blue

**DOI:** 10.1186/s11671-025-04424-2

**Published:** 2026-01-19

**Authors:** Tooba, Nasir Assad, Marzia Batool Laila, Rao Muhammad Faisal Iqbal, Laiba Manahil, Jamshed Iqbal, Muhammad Naeem-ul-Hassan, Anusha Khuram, Yasir Assad, Muhammad Nauman Khan, Shabab Hussain, Alevcan Kaplan, Amal M. Al-Mohaimeed, Islem Abid

**Affiliations:** 1 Institute of Chemistry, University of Sargodha, Sargodha, 40100 Punjab Pakistan; 2 Department of Chemistry, The University of Lahore, Sargodha Campus, 10 Km Lahore Road, Sargodha, Punjab Pakistan; 3 Department of Chemistry and Industrial Chemistry, University of Genoa, Via Dodecaneso, 31, 16146 Genoa, Italy; 4 Department of Zoology, University of Sargodha, Sargodha, 40100 Punjab Pakistan; 5 Department of Zoology, Hazara University Mansehra, Mansehra, Khyber Pakhtunkhwa Pakistan; 6 Department of Botany, University of Chakwal, Punjab, Pakistan; 7 Department of Biomedical and Clinical Sciences, University of Milan, 20157 Milan, Italy; 8 Department of Pharmaceutical Botany, Faculty of Pharmacy, Dicle University, 21200 Diyarbakır, Turkey; 9 Department of Chemistry, College of Science, King Saud University, P.O. Box 22452, 11495 Riyadh, Saudi Arabia; 10 Center of Excellence in Biotechnology Research (CEBR), DSR, King Saud University, P.O. 2455, 11495 Riyadh, Saudi Arabia

**Keywords:** Green synthesis, Silver nanoparticles, *Solanum lycopersicum*, Colorimetric sensing, Dye degradation

## Abstract

This study reports the green synthesis of silver nanoparticles (AgNPs) using *Solanum lycopersicum* var. cerasiforme (SLC) leaves extract as a natural reducing and stabilizing agent under direct sunlight for just only 3 min. The main objective was to develop a rapid, cost-effective, and environmentally friendly method to fabricate SLC-functionalized AgNPs (SLC-AgNPs) for dual applications: highly selective colorimetric detection of mercury ions (Hg^2^⁺) and photocatalytic degradation of methylene blue (MB) dye. The synthesized nanoparticles were characterized by UV–Vis, FTIR, XRD, SEM, EDX, and DLS, confirming their spherical shape (~ 38 nm), crystalline structure, and stable surface functionalization. The SLC-AgNPs exhibited exceptional selectivity for Hg^2^⁺ through a redox reaction mechanism, enabling colorimetric sensing with a low detection limit of 37.7 nM and a linear response range of 40–180 nM. Detection of Hg^2^⁺ in real river and tap water samples validated the sensor’s practical applicability, with recoveries above 85%. In addition to sensing, the SLC-AgNPs demonstrated significant photocatalytic efficiency, degrading 83.4% of MB dye within 80 min of sunlight exposure, following pseudo-first-order kinetics with an activation energy of 35.02 kJ/mol. This dual- functionality capability highlights the novelty of the green synthesized SLC-AgNPs as an eco-friendly nanomaterial that combines sensitive heavy metal detection with effective dye degradation. These findings suggest promising potential for SLC-AgNPs in sustainable environmental monitoring and wastewater treatment, bridging cost-efficiency with high-performance nanotechnology.

**Supplementary Information:**

The online version contains supplementary material available at 10.1186/s11671-025-04424-2.

## Introduction

Global attention and popularization are focused on nanotechnology research, which remains the foundation for numerous significant advances and achievements, and will probably continue to do so in the future, along with other remarkable scientific findings [1]; [2]. The relevance of nanoscience and its rapid applications, research, and development in this field have exploded globally [3]. The remarkable properties of nanomaterials, including size, shape, topography, solubility, and other physicochemical features, have enabled tremendous advancement in nanoscience [4]; [5]. The physical characteristics of nanoparticles (NPs) vary substantially from those of their source material due to their large surface area and small size [6]; [7]. This technology involves the production of nanomaterials, used in different fields, such as catalysis, heavy metal sensing, antimicrobial agents, and biomedicine [8]; [9]; [10] [11].

NPs are synthesized using various physical and chemical techniques; nevertheless, these approaches are not eco-friendly and are costly [12]; [13]. Biological methods have been proposed as a viable alternative for synthesizing NPs to meet current demand [14]. In NP production plants, fungi, algae, and bacteria are explored. Using In this case, employing plant materials for NP synthesis is more advantageous than other methods because of its eco-friendly nature and cost-effectiveness. Moreover, plant-mediated synthesis is generally faster, more cost-effective, and simpler to scale up for producing substantial quantities of NPs [15]; [16]. Silver nanoparticles(AgNPs) are sought after for advanced applications in all areas of technology and study [17]. Compared to traditional methods, green chemistry offers many advantages for fabricating AgNPs [18]. These advantages include reduced energy consumption, straightforward scalability for large-scale NP production, higher yields, cost-effectiveness, and no need for specialized isolation or culture preparation. Furthermore, it is environmentally sustainable [19]. Because they lack harmful chemical compounds on their surface, AgNPs are safe for biological cell types and useful in sensing and dye degradation applications [20]. This is accomplished through the reduction and stabilization of these elements utilizing plant extracts [21, 22]. Several plant extracts, each with its a unique composition, such as those from *Pistacia atlantica* Desf. [23]*, Catharanthus roseus* (L.) G.Don [24]*, Citrullus lanatus* (Thunb.) Matsum. & Nakai [25]*, Myrsine africana* L. leaves [26], and *Olea europaea* L. leaves [9]*,* have been identified and documented for their role in the green synthesis of AgNPs.

Four heavy metals lead, cadmium, mercury, and arsenic, are among the 10 hazardous compounds or groups of chemicals that the World Health Organization (WHO) highlighted in 2017, along with other health concerns. Mercury tops the list and is a notorious toxicant [27]. Because of its high solubility in water, the water-soluble divalent mercuric ion (Hg^2+^) is one of the most prevalent and stable inorganic mercury forms. It has a density of 13.53 g cm^−3^ and does not float on the surface of water [28]. Mercury (Hg^2+^) is an omnipresent environmental danger with both natural and anthropogenic origins [29]. Hg^2+^ has harmful effects on the central nervous system, kidneys, endocrine system, and brain [30]. It is important to monitor Hg^2+^ levels in the environment under aqueous conditions with high sensitivity and selectivity to reduce interference from other metal ions. Traditional analytical techniques, such as Inductively Coupled Plasma Mass Spectrometry (ICP-MS) and Atomic Absorption Spectrometry (AAS), for detecting heavy metals involve expensive, time-consuming sample preparation, complex instruments, and sophisticated sample handling [31]. Thus, easy, quick, and cost-effective techniques for tracking heavy metal contamination in diverse matrices are highly desirable. Colorimetric sensors have attracted significant attention because of their inexpensiveness, ease of use, and capacity to deliver quick and clear data for the identification of toxic metals [32]. In this regard, plasmonic metal nanoparticles such as AgNPs have garnered much interest because of their remarkable optical properties resulting from localized surface plasmon resonance (LSPR). This phenomenon is responsible for their unique coloration, which varies with changes in wavelength and absorption intensity [33]. These features make them attractive options for the development of colorimetric sensors. Therefore, AgNPs synthesized using SLC extract were used for the colorimetric detection of Hg^2^⁺.

On the other hand, organic dyes are believed to contain carcinogenic and mutagenic ingredients, making them among the most dangerous pollutants [34]. These substances exhibit significant resistance to degradation, allowing them to persist in the environment for prolonged durations [35]. Consequently, several health problems and numerous environmental hazards have been associated with these poisonous dyes. These include kidney and liver damage, toxicity affecting the central nervous system, skin illnesses, and various blood abnormalities [36]. Methylene blue (MB) has a robust heterocyclic aromatic structure with a central sulfur atom. Because of its structure, it is more difficult to break down naturally by bacteria or sunlight [37]. Consequently, it is essential for MB dye in effluents to undergo degradation to mitigate its detrimental impacts. Dye degradation processes include various physical and conventional procedures, such as activated carbon sorption, flocculation, electrocoagulation, ultraviolet light degradation, and redox treatments [38]. Ozonation is a common approach for dye deterioration. However, these methods often require significant energy, incur high costs, and generate unwanted by-products [39]. As a result, an environmentally safe method needs to be developed. SLC-AgNPs have unique physicochemical and electrical properties not found in bulk materials, making them a viable alternative to conventional dye degradation techniques [40].

Herein we synthesized an easy-to-use, cost-effective, and highly selective colorimetric Hg^2+^ sensor based on Beefsteak tomato (*Solanum lycopersicum var. cerasiforme* (SLC) leaves extract-functionalized silver nanoparticles (SLC-AgNPs). The green synthesized SLC-AgNPs were characterized by UV–visible spectrophotometry, Fourier-Transform Infrared Spectroscopy (FTIR), X-ray diffraction (XRD), Scanning Electron Microscopy (SEM), Energy Dispersive Spectroscopy (EDX) and DLS (Dynamic Light Scattering). The colorimetric sensing ability of SLC-AgNPs was evaluated against Hg^2+^ in aqueous solution among various competing transition metal ions. The feasibility of detecting Hg^2+^ was also assessed in river water and tap water samples. Additionally, the effectiveness of SLC-AgNPs was evaluated for photocatalytic degradation of MB dye. Several plant species have been successfully utilized in the green synthesis of AgNPs due to their rich phytochemical content, which acts as natural reducing and capping agents. However, this study introduces a novel and rapid sunlight-assisted synthesis approach using *S lycopersicum* leaves extract. Unlike many previous methods that require long reaction times, additional heating, or pH adjustment, our approach achieves stable AgNP formation within just 3 min of natural sunlight exposure. Moreover, the use of *S. lycopersicum*, a commonly available plant along with a rich phytochemical composition (e.g., flavonoids, alkaloids, phenolics), adds novelty to the synthesis route [41]. This method is not only eco-friendly and cost-effective but also highly efficient in producing AgNPs with dual functionality: colorimetric detection of Hg^2^⁺ and photocatalytic degradation of dyes, demonstrating enhanced multifunctionality over previously reported systems.

## Experimental and methodology

### Materials

Silver nitrate (AgNO_3_ ≥ 99%), (Hg(NO_3_)_2_ 99%), (CdNO_3_ ≥ 99%), (CoNO_3_ ≥ 98%), (PbNO_3_ ≥ 99%), (NiNO_3_ ≥ 99%), (FeCl_3_ ≥ 98%), were purchased from Sigma-Aldrich. All the reagents used in the experiment were of analytical grade. Throughout the experiment, solutions were prepared using deionized water (DW). *S. lycopersicum* (SLC) leaves were collected from privately owned land belonging to one of the authors in Sargodha District, Punjab, Pakistan, and identified, by Dr. Muhammad Nauman Khan, Department of Botany, Islamia College Peshawar, Peshawar 25120, Pakistan. The species is not protected, and is not listed as endangered. Therefore, no specific permissions or licenses were required for its collection. All procedures complied with relevant institutional, national, and international guidelines and legislation.

### Extraction of SLC

A standardized method was employed to prepare an aqueous extract of SLC leaves [42]. Fresh SLC leaves were thoroughly rinsed with distilled water to eliminate dirt and other contaminants. The samples were then dried in the shade for eight days. After drying, the SLC leaves were coarsely ground into a fine powder, which was passed through a sieve to remove larger particles. 5 g powder were mixed with 200 mL of distilled water, using a magnetic stirrer for 2.5 h at 50 °C. The suspension was then filtered with Whatman filter paper No. 41, and the filtrate was collected in a conical flask and stored at 4 °C for further analysis [43].

### Synthesis of green silver nanoparticles using SLC extract (SLC-AgNPs)

**Fig. 1:**
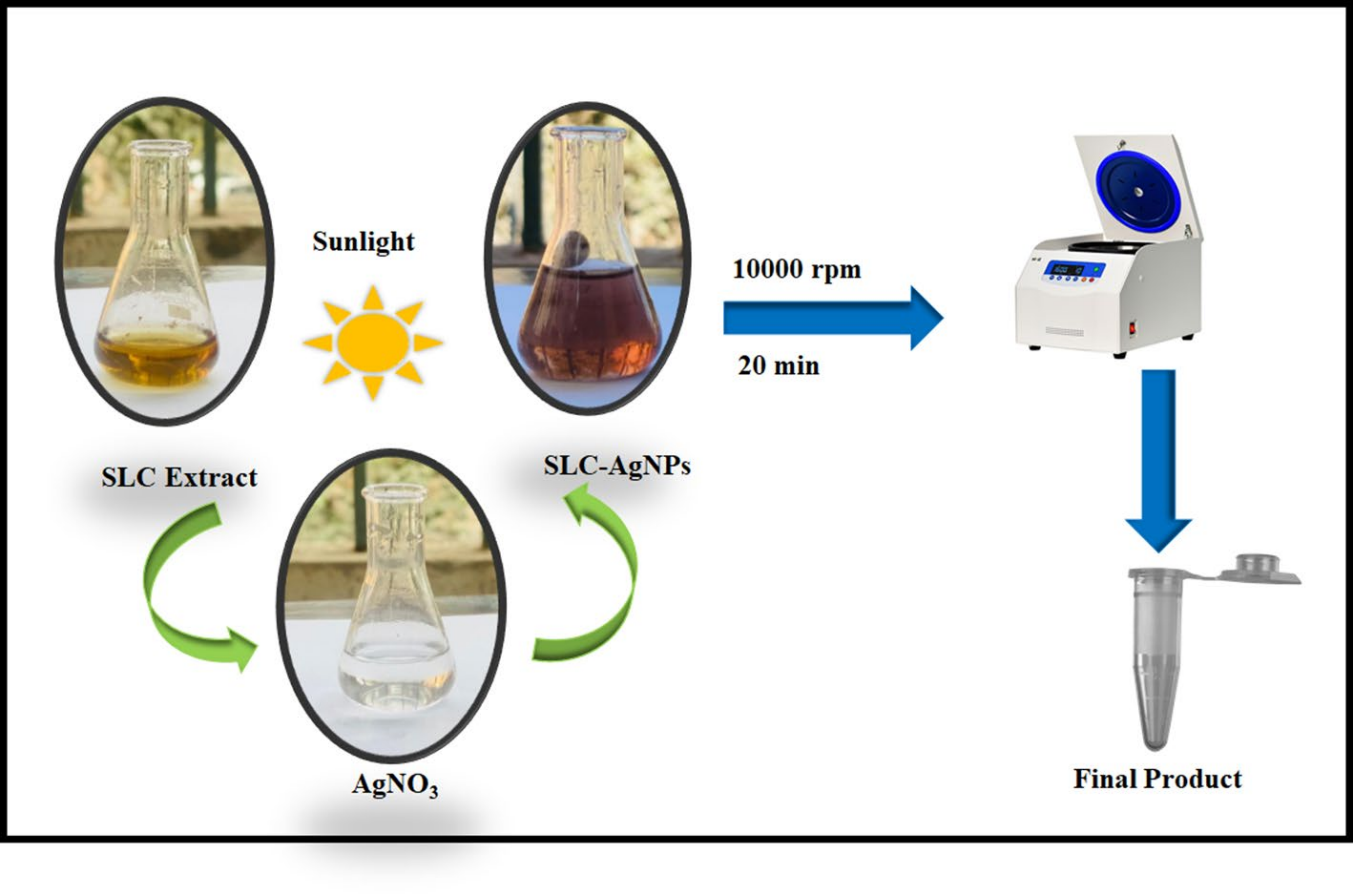
Schematic representation of the green synthesis of silver nanoparticles (SLC-AgNPs) using SLC extract and AgNO₃ under sunlight exposure

For the synthesis of SLC-AgNPs, 10 mL of liquid extract was mixed with 10 mL of a 1.0 mM AgNO_3_ solution, following the protocol of Assad et al., (2023) with some modifications [44]. The mixture was then placed outdoors for different durations on bright, sunny days, and exposed to direct sunlight, with regular observations to monitor the reaction‘s progress. All sunlight-assisted synthesis experiments were performed on clear, sunny days between 10:00 AM and 2:00 PM under direct solar exposure (UV to IR spectrum). The UV index was monitored using a mobile application and maintained between 7 and 10 to ensure consistent irradiation conditions. This ensured consistent and effective photoreduction of Ag⁺ ions using *S. lycopersicum* leaf extract. As the concentration of AgNPs increased, the solution changed color from yellowish brown to a deeper brown. The solution was then centrifuged for 20 min at 10,000 rpm. The washing procedure was performed three times to remove the unreacted material. A portion of the isolated pure solid AgNPs was utilized for characterization analysis, as shown in Fig. 1. The impact of various synthesis parameters on the preparation of SLC-AgNPs was investigated. The influence of SLC concentration on the production of SLC-AgNPs was examined by incorporating 1–20 mL of SLC extract into the synthesis process alongside 10 mL of 1 mM AgNO_3_ solution. The reaction mixture was then subjected to sunlight for 3 min, during which LSPR was assessed using UV–vis spectra. The impact of different concentrations of Ag^+^ ions (from 1 to 5 mM) on the synthesis of SLC-AgNPs using 10 mL of SLC was investigated. After mixing, the solution was subjected to sunlight exposure for about 3 min. Similarly, a 1 mM (10 mL) Ag^+^ solution was mixed with SLC (1 mg/10 mL) to investigate the effect of sunlight exposure on the synthesis of SLC-AgNPs utilizing SLC extract. The exposure duration to sunlight varied between 10 s and 3 min. Moreever, the stability of the biosynthesized SLC-AgNPs was evaluated under different pH conditions.

### Effect of different physiochemical parameters on the synthesis of SLC-AgNPs

To find the best reaction conditions, the current study investigated several physicochemical parameters affecting SLC-AgNP synthesis. Experimental variables such as plant extract dose, Ag^+^ ion concentration, and reaction duration can influence AgNP synthesis. UV–vis spectroscopy was employed to record and identify the ideal conditions for SLC-AgNPs production.

#### Characterization

The biosynthesized SLC-AgNPs were characterized by UV–visible spectrophotometry, Fourier-Transform Infrared Spectroscopy (FTIR), X-ray diffraction (XRD), Scanning Electron Microscopy (SEM), Energy Dispersive Spectroscopy (EDX) and DLS (|Dynamic Light Scatering). The absorption spectra of SLC and SLC-AgNPs were obtained using a UV–Vis spectrophotometer (UV-1800 Shimadzu) over the 200–800 nm range. The SLC extract and SLC-capped AgNPs were analyzed by FTIR spectroscopy with a Shimadzu FTIR-8400S spectrometer using the KBr pellet method. Both spectra were derived from 20 scans at a resolution of 2 cm^−1^, covering the range of 4000–400 cm^−1^, to improve the signal-to-noise ratio. Crystal size and phase of the biosynthesized SLC-AgNPs were evaluated by XRD (JDX-3532, JEOL, Tokyo, Japan). The surface morphology, dimensions, structure, elemental composition, and quantification of SLC-AgNPs were analyzed using EDX (Carbon Sticker No G3347,) Plano, Wetzlar, Germany) alongside scanning electron microscopy (SEM) (Carbon Sticker No G3347) Plano (Wetzlar, Germany). Images were produced utilizing SEM–EDX with an acceleration voltage of 5 kV. A chemical microanalysis was conducted utilizing SEM–EDX techniques. The sample was meticulously drop-cast onto a pristine glassy carbon plate for SEM measurements, followed by drying before imaging. Zeta potential (ZP) and Zetasizer (Malvern Zetasizer Nano ZS) were utilized to assess zeta potential and perform DLS analysis. The topological charge and resilience of SLC-AgNPs were evaluated using ZP, and the size distribution of the synthesized SLC-AgNPs was determined by DLS.

#### Colorimetric sensing of heavy metal ions using SLC-AgNPs

The biosynthesized SLC-Ag NPs were combined with various metal ion solutions (Cd^2+^, Co^2+^, Pb^2+^, Ni^2+^, Hg^2+^, Fe^2+^, Zn^2+^, and Mn^2+^) to evaluate the potential sensing ability of the SLC-AgNPs, following the protocol of Jabbar et al., (2023) with minor modifications [20]. Test tubes were prepared, each holding a suspension of SLC-AgNPs (1 mL) mixed with different metal ion solution (1 mL of 1 mM) for this experiment. The UV–vis spectrum of each solution containing SLC-Ag NPs and metal ions was then recorded. A notable color change was detected for Hg^2+^. The minimum concentration of Hg^2+^ detectable with the SLC-Ag NPs was determined by analyzing the spectra across a range of Hg^2+^ concentrations from 40 to 180 nano molar (nM) and constructing a calibration curve.

#### Photocatalytic degradation of methyl blue using SLC-AgNPs

The photocatalytic performance of biosynthesized SLC-AgNPs was assessed by degrading MB dye under sunlight, following the protocol specified by Assad et al., (2024), with few modifications [45]. Briefly 5 mg of was dissolved MB in 500 mL of distilled water to prepare a stock solution. Then, 5 mg of SLC-AgNPs was added to 30 mL of the dye solution, which was continuously stirred at 150 rpm and then incubated in the dark for 20 min. After incubation, the mixture was exposed to sunlight for 80 min, and absorption spectra were taken at various intervals during this period. The photocatalytic degradation experiments were performed under direct sunlight between 10:00 AM and 2:00 PM on clear, sunny days. The sunlight intensity during the experiments was monitored using a mobile application, with the UV index ranging from 7 to 10, ensuring consistent full-spectrum irradiation (UV to IR) across all trials. The gradual fading of color in the solutions indicated dye degradation. Equation (1) was used to calculate the percentage of dye degradation in each solution.

1 $$ {{\% dye degradation }} = { }\frac{{A_{0} - A_{f} }}{{A_{0} }} \times 100 $$

This is where the absorbance of the dye at various intervals (A_f_) is shown alongside its initial absorbance (A_0_). The peak, absorbance of dye MB was recorded at 665 nm.

### Statistical analysis

All experiments were performed in triplicate, and results are expressed as mean ± standard deviation (SD). To evaluate the significance of differences across experimental groups, a one-way ANOVA was applied using Origin 2021 software. Statistical significance was considered at *p* < 0.05.

## Results and discussion

### Physicochemical characterization of the synthesized SLC-AgNPs

#### SLC-AgNPs synthesis and potential mechanisms of synthesis

**Fig. 2:**
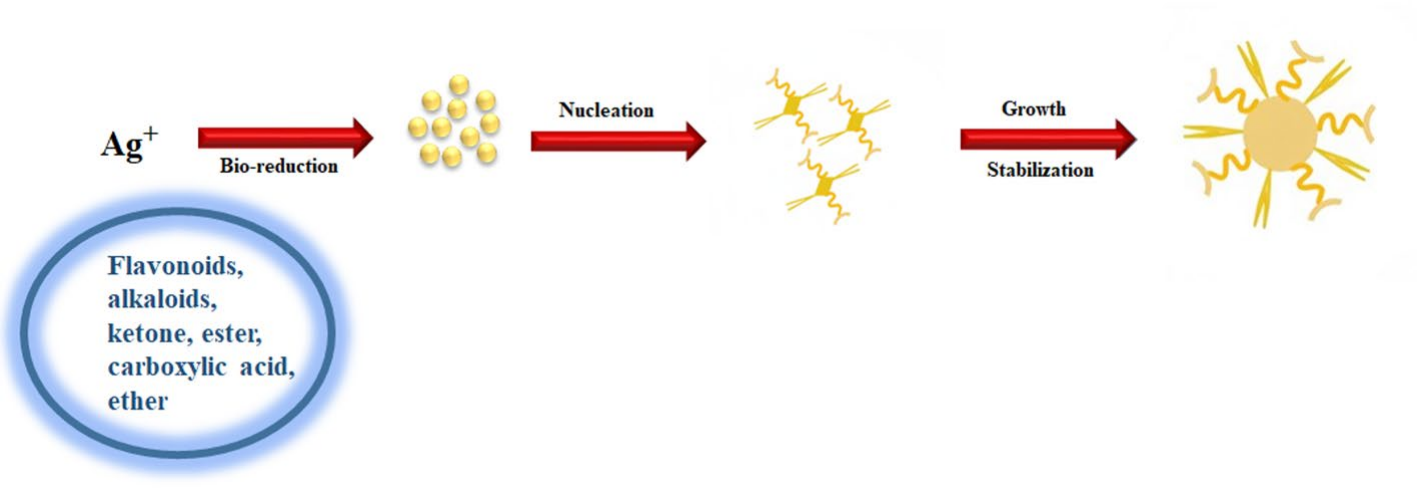
Synthesis scheme for biosynthesized SLC-AgNPs

Using the green approach, yellowish-brown SLC-AgNPs were synthesized by mixing SLC extract with AgNO_3_, and solution, exposing the mixture to sunlight for 3 min. Figure 2 shows the synthesis scheme for SLC-AgNPs. The SLC leaves extract, AgNO_3_ solution, and the synthesized yellowish-brown SLC-AgNPs are shown in Fig. 1. The phytochemicals in the SLC leaves extract reduced Ag^+^ ions to metallic Ag^0^, which subsequently aggregated to form yellowish-brown SLC-AgNPs. The phytochemicals in the extract of SLC leaf extract eliminated the need for potentially hazardous reducing agents by acting as both reducing and capping agents. The mechanism for SLC-AgNPs synthesis involves the reduction of Ag^+^ ions to Ag^0^ by the phytochemicals in the SLC leaves extract upon exposure to solar radiation, as illustrated in Fig. 2. The active phytochemical compounds in SLC extracts, such as monoterpenes, phenolics, flavonoids, alkaloids, sterols, and other substances like ketones, esters, carboxylic acids, ether, and polyphenols, reduce Ag^+^ in AgNO_3_ to generate Ag^0^ (Eq. 2) [46]. No additional toxic reducing agents or capping agents were used in this process because the phytochemicals in SLC served as stabilizing, ameliorating, and reducing agents [47]. The mechanism is summarized as follows;

2 $$\mathrm{Ag}N{O}_{3} +\text{ SLC}-\text{X }\to \left(\mathrm{Ag}N{O}_{3}\right)-\left(\mathrm{SLC}-\text{X }\right) \to A{g}^{0} + {N{O}_{3}}^{-} +\text{ Oxidation Products}$$

where “X” denotes the ester, ketone, carboxylic acid, and ether groups in the SLC extract.

In the nucleation process, which follows the conversion of Ag^+^ to Ag^0^ atoms, Ag^0^ atoms ultimately clump together to form nanoparticles. These phytochemicals initiate SLC binding on these clusters. The functional groups on these incorporated aggregates further transform Ag^+^ atoms into Ag^0^ atoms (Eq. 3):

3 $${A{g}^{0}}_{m} + 1(\text{ SLC}-\mathrm{X}) \to ({A{g}^{0}}_{m}) -{\left(\mathrm{SLC}-\mathrm{X}\right)}_{1}$$

These phytochemicals in the SLC extract promote growth and stabilization by collision, Ag^+^ fusion, and growth, resulting in the production of AgNPs. The Fig. 2 presents the detailed mechanisms involved in the synthesis of SLC-AgNPs. The nucleation and growth processes determine the size and shape of any metal nanostructure, and these processes are affected by the quantity and composition of active species in the extract. Nucleation refers to the reduction of metal ions in bulk, whereas growth describes the reduction of metal ions during the nucleus stage [48].

#### Effect of concentration of SLC on AgNPs synthesis

**Fig. 3:**
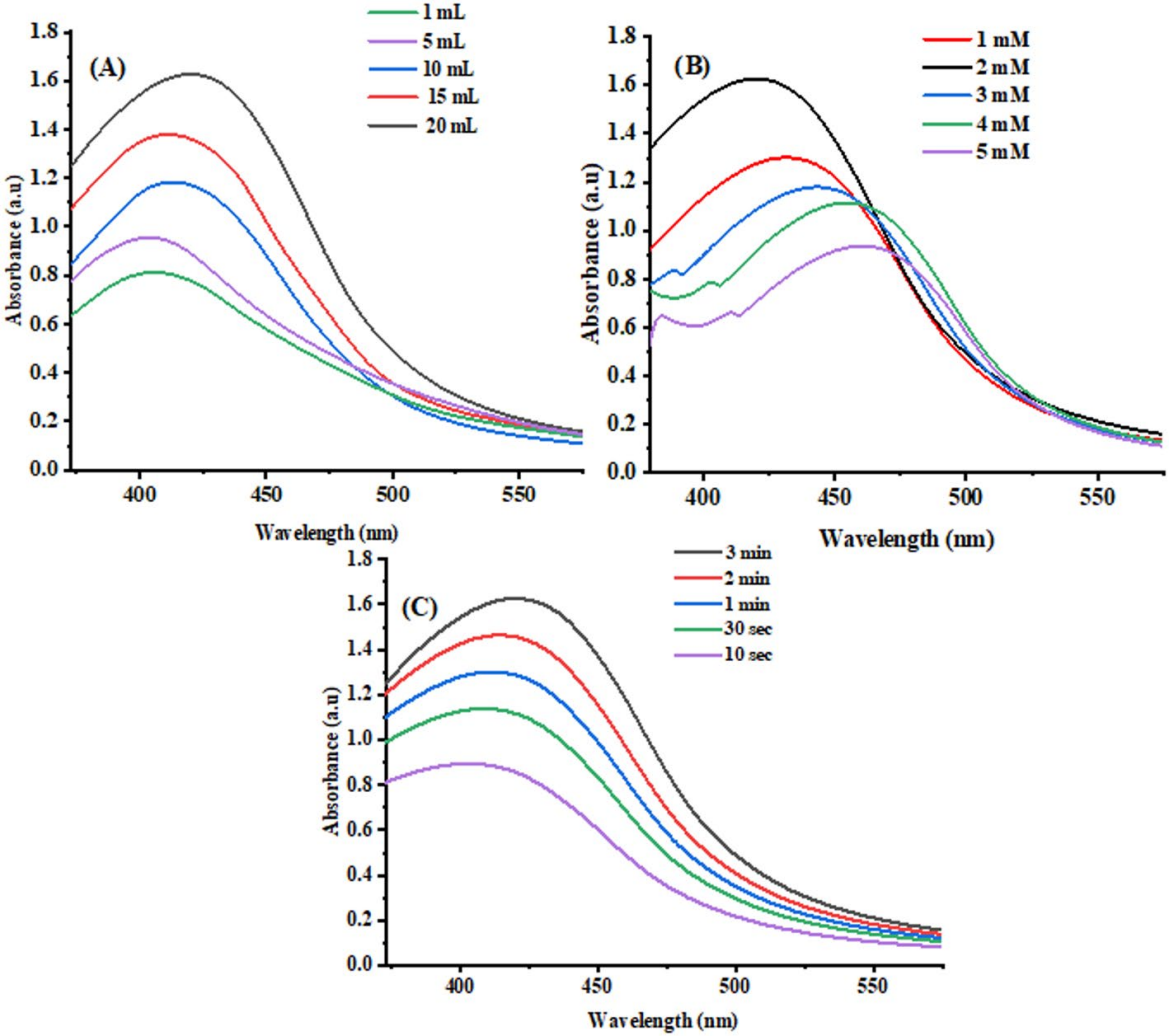
**A** Effect of SLC extract on SLC-AgNPs, **B** Effect of Ag^+^ concentration on SLC-AgNPs, **C** Effect of exposure to sun radiation on SLC-AgNPs

To study the effect of SLC extract on SLC-AgNP production, a solution containing SLC extract (1–20 mL) and 1 mM Ag^+^ ions (10 mL) was subjected to sunlight for a predetermined duration. The UV–vis spectra of each sample were documented. A unique localized surface plasmon resonance (LSPR) peak for AgNPs was observed in all samples as the SLC extract dose increased from 1 to 20 mL. A bathochromic shift in absorbance and a movement toward a greater λ_max_ occurred as the SLC dose increased from 1 to 20 mL. For SLC dosages of 1, 5, 10, 15, and 20 mL, the λ_max_ values were 398, 403, 407, 411, and 420 nm, respectively. This red shift in λ_max_ is attributed to the presence of fewer functional groups in the leaf extract when 1 mL of extract was combined with 1 mM Ag^+^ ions. As a result, fewer and smaller-sized SLC-AgNPs were produced, resulting in a shorter λ_max_ and lower absorbance of the LSPR peak. At the same Ag^+^ ion concentration, increasing the SLC extract dosage made more functional groups accessible, leading to the formation of bigger AgNPs and a change in λ_max_ to a higher wavelength with higher absorbance, as shown in Fig. 3A.

#### Effect of Ag^+^ ions concentration on AgNPs synthesis

The effects of different concentrations of Ag^+^ ions on the production of SLC-AgNPs were studied by mixing 10 mL of SLC extract with 10 mL of Ag^+^ ions at concentrations ranging from 1 to 5 mM, then exposing the mixture to sunlight for 3 min. Figure 3B displayed the UV–vis spectra recorded for each AgNPs sample. As the concentration of Ag^+^ ions increased from 1 to 5 mM, a distinct LSPR peak for AgNPs appeared in each sample. Increasing the Ag + ion concentration from 1 to 5 mM caused a hypochromic shift in absorbance, with the λmax moving to higher wavelengths. The λmax values for the 1, 2, 3, 4, and 5 mM Ag^+^ ion solutions were 420, 433, 440, 446, and 451 nm, respectively. This red shift in λ_max_ occurs because, in the 1 mM Ag^+^ ion solution mixed with 10 mL of SLC extract, there were sufficient ions to form a specific number of smaller SLC-AgNPs, resulting in a shorter λ_max_ for the LSPR peak.

#### Effect of sunlight exposure time on SLC-AgNPs synthesis

SLC-AgNPs were prepared by mixing 10 mL of SLC extract with 10 mL of Ag^+^ ion (1 mM) solution, and the mixture was then exposed to sunlight for 10, 30, 1, 2, 3, and min. This parameter investigated the effects of sunlight exposure on the synthesis of SLC-AgNPs. The UV–vis spectrum was recorded at regular time intervals. At each time interval (from 10 s to 3 min), SLC-AgNPs exhibited a distinct LSPR peak. As the duration of sunlight exposure increased-from 10 s to 3 min the λ_max_ shifted to a higher wavelength with a hypsochromic shift in absorbance. The λ_max_ values were 402, 408, 411, 414, and 420 nm at 10 s, 1 min, 2 min, and 3 min, respectively. The smaller-sized SLC-AgNPs present throughout the 10 s exposure period produced a red shift in λ_max_ by shortening the LSPR peak. As the exposure duration increased, more AgNPs with larger sizes were produced, causing the λ_max_ to shift to a higher wavelength with higher absorbance. As shown in Fig. 3C, the UV–vis spectra of SLC-AgNPs were affected by exposure to sunlight.

#### Effect of pH on the stability of SLC-AgNPs in terms of LSPR

**Fig. 4:**
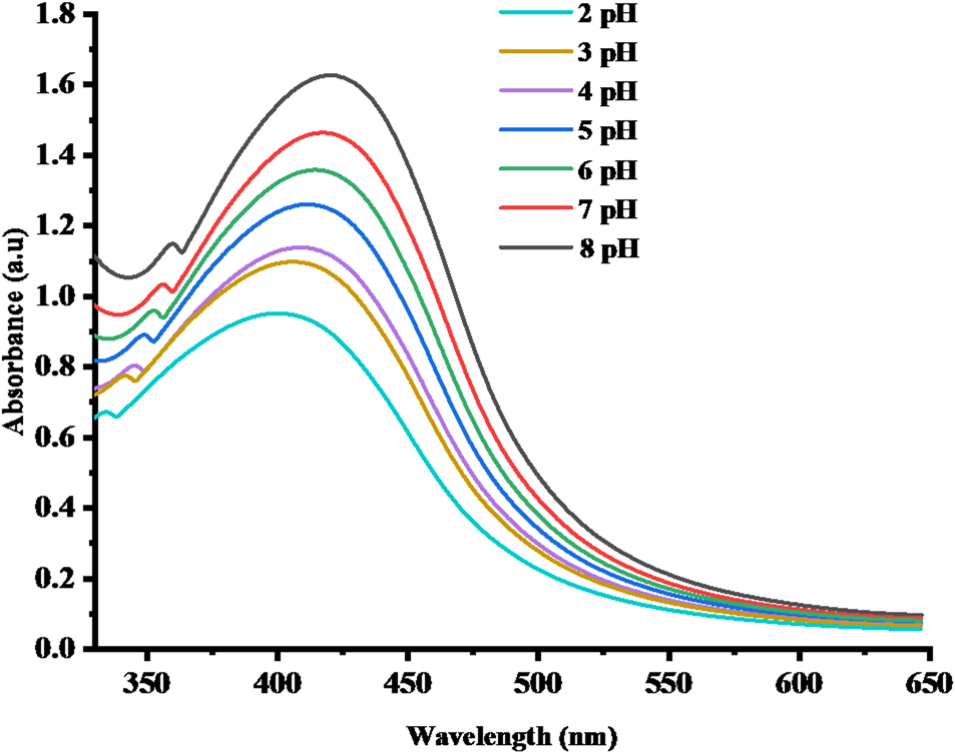
Effect of pH on biosynthesized SLC-AgNPs

The surface charge of nanoparticles (NPs) varies in response to variations in the pH of the dispersion medium. This charge shift allows for the prediction of NPs stability in a given dispersion medium. The stability of SLC-AgNPs in dispersion media was evaluated by dispersing them in a pH range of 2 to 8, and recording their UV–vis spectra. Figure 4 presents these UV–vis spectra. As shown in Fig. 4, absorbance was lower at acidic pH values and increased as the pH shifted from 5 toward neutral and alkaline conditions. Maximum absorption was observed at pH 8. This is because, at acidic pH (pH ≤ 4), protons in the dispersion media neutralized the negative charges on the SLC-AgNPs surface, as shown by the SLC-AgNPs zeta potential of 23.7 ± 2.4 mV. Because of this, the AgNPs aggregated, reducing their absorption and, ultimately, their stability. The stability of the suspension is maintained by the mutual repulsion of the negatively charged NPs, and the functional groups of the AgNPs do not protonate when the dispersion medium pH is above 5. Increased repulsion between the NPs increases the stability of smaller-sized NPs at alkaline pH levels. This increased the absorption intensity, which in turn raised the stability of SLC-AgNPs. For medical applications, the produced NPs must be stable at physiological pH.

One important consideration when using NPs for practical applications is their colloidal stability [49]. Producing stabilized NPs requires the use of stabilizing chemicals because uncapped NPs have high surface energy and a tendency to aggregate. In this work, SLC was used to stabilize AgNPs. Because of the repulsive forces between individual NPs, the resulting SLC-AgNPs have a negative surface charge and exhibit good stability in the liquid phase, as shown by their negative zeta potential value of -23.7 ± 2.4 mV. The nitrogen containing groups in the SLC are primarily responsible for the negative surface charge. The temperature, pH of the medium, and electrolytic ion concentration all significantly affect the colloidal stability of AgNPs. The aggregation of NPs occurred when the electrolyte neutralized the charges on their surfaces. Furthermore, as Fig. 4 illustrates, the stability of the NPs was evaluated over a wide pH range (2–8). The UV–vis spectrum in an alkaline electrolyte was not significantly altered, suggesting that deprotonation of the amine functionalities on the surface of SLC-AgNPs contributed to the high stability of NPs in an alkaline environment. On the other hand, a notable reduction in absorption intensity and, consequently, the stability of the SLC-AgNPs was noted in the acidic medium. This might be because acid protons neutralized the NPs’ surface charges, reducing their repulsion and causing aggregation.

### Characterization

**Fig. 5:**
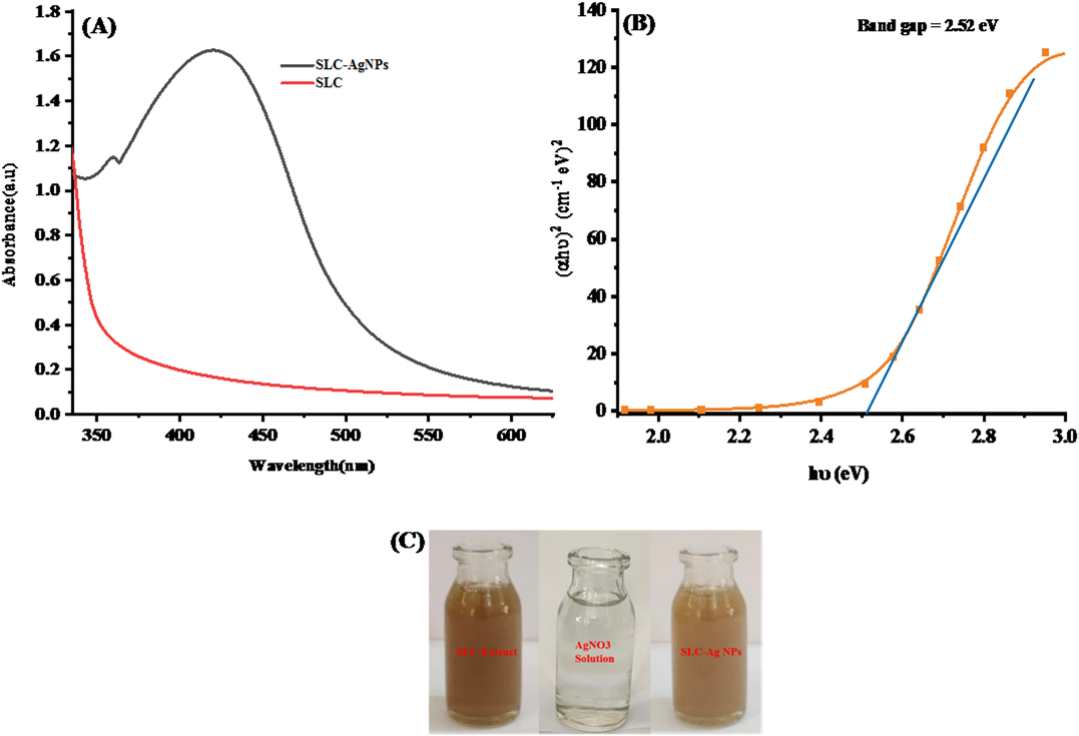
**A** UV–vis spectrum of biogenic SLC-AgNPs and SLC extract, **B** The determination of the optical band gap (E_g_) for SLC-AgNPs

SLC leaves extract mediated biosynthesis of AgNPs was exposed to sunlight, resulting in a color change from yellow to brown. This color change indicated the formation of SLC-AgNPs. The bioactive compounds present in the SLC extract acted as both reducing and capping agents. A UV–vis spectral peak at 420 nm further supported the LSPR of AgNPs, as shown in Fig. 5A. Our results were in good agreement with those of Assad et al., (2023) [44]

The bandgap of SLC-AgNPs was calculated using data collected from UV–visible spectra using a tauc plot (Eq. 4).

4 $$ \left( {\alpha h\nu } \right)^{n} = A\left( {h\nu - E_{g} } \right) $$

The absorption coefficient, Planck‘s constant, frequency, energy-independent constant, bandgap energy, and type of transition (*n* = 2 for direct band gap material) are denoted by α, h, $\nu$, A, E_g_, and n, respectively. The term "h $\nu$" represents the incident photon energy. In the Tauc plot, the x-axis is labeled "h $\nu$" and the y-axis is labeled "($\alpha h\nu$)^n^". The bandgap value of 2.52 eV was determined from the linear relationship between ($\alpha h\nu$)^2^ and $h\nu$ as shown in Fig. 5B. Previously reported energy bandgap values for AgNPs are consistent with this result. Mistry et al., (2021) reported a band gap energy of 2.48 eV for AgNPs [50]. Aziz et al., (2018) found that AgNPs synthesized by the sol–gel method have a band gap energy of 2.5 eV [51]. The measured bandgap of 2.52 eV indicates that the synthesized SLC-AgNPs possess photocatalytic characteristics.

**Fig. 6:**
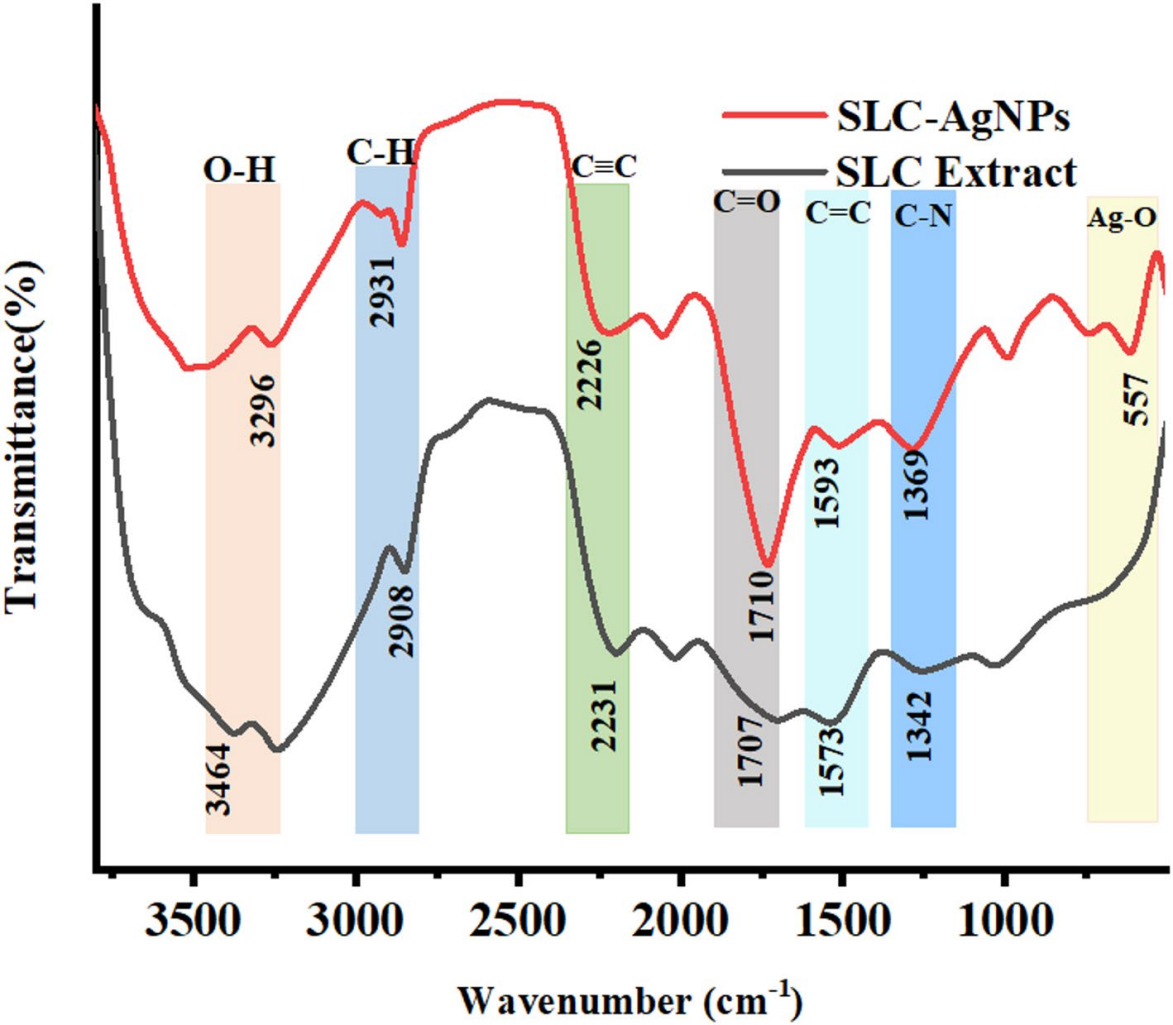
FTIR spectra of biosynthesized SLC-AgNPs and SLC extract

The biomolecules and functional groups present in the SLC extract and SLC-AgNPs were evaluated using FTIR spectra ranging from 400 to 4000 cm^− 1^. The results demonstrated that phytochemicals are involved in the biosynthesis of SLC-AgNPs. The spectrum of the SLC extract and SLC-AgNPs, showed little difference in peak positions of the peaks as shown in Fig. 6. The shifts in functional groups between the SLC extract and SLC-AgNPs in the FTIR spectra provide evidence that SLC-AgNPs biosynthesis occurs successfully. Since phytochemicals facilitate the reduction of stable Ag⁺ to Ag⁰, this is reflected in the shifts of functional groups. The broad O–H/N–H stretching peak shifting from 3464 cm⁻^1^ to 3296 cm⁻^1^ emphasizes that hydroxyl and amine groups are primarily responsible for stabilizing the NPs [21];[52]. The shift in the C = O stretching peak from 1707 cm⁻^1^ to 1710 cm⁻^1^ indicates is the participation of carbonyl groups in capping of the nanoparticles [17]. The shift in the aromatic C = C stretching from 1573 cm⁻^1^ to 1593 cm⁻^1^ further confirms the involvement of active polyphenolic compounds in nanoparticles formation [20]. The IR absorption peak at 557 cm⁻^1^, characteristic of Ag–O bonds, which is absent in the extract but present in the SLC-AgNPs, confirms that the Ag nanoparticles are firmly connected with the bioactive compounds validating the synthesis of stable AgNPs [9];[53]. These findings support the synthesis of SLC-AgNPs using SLC extract through eco -friendly methods.

**Fig. 7:**
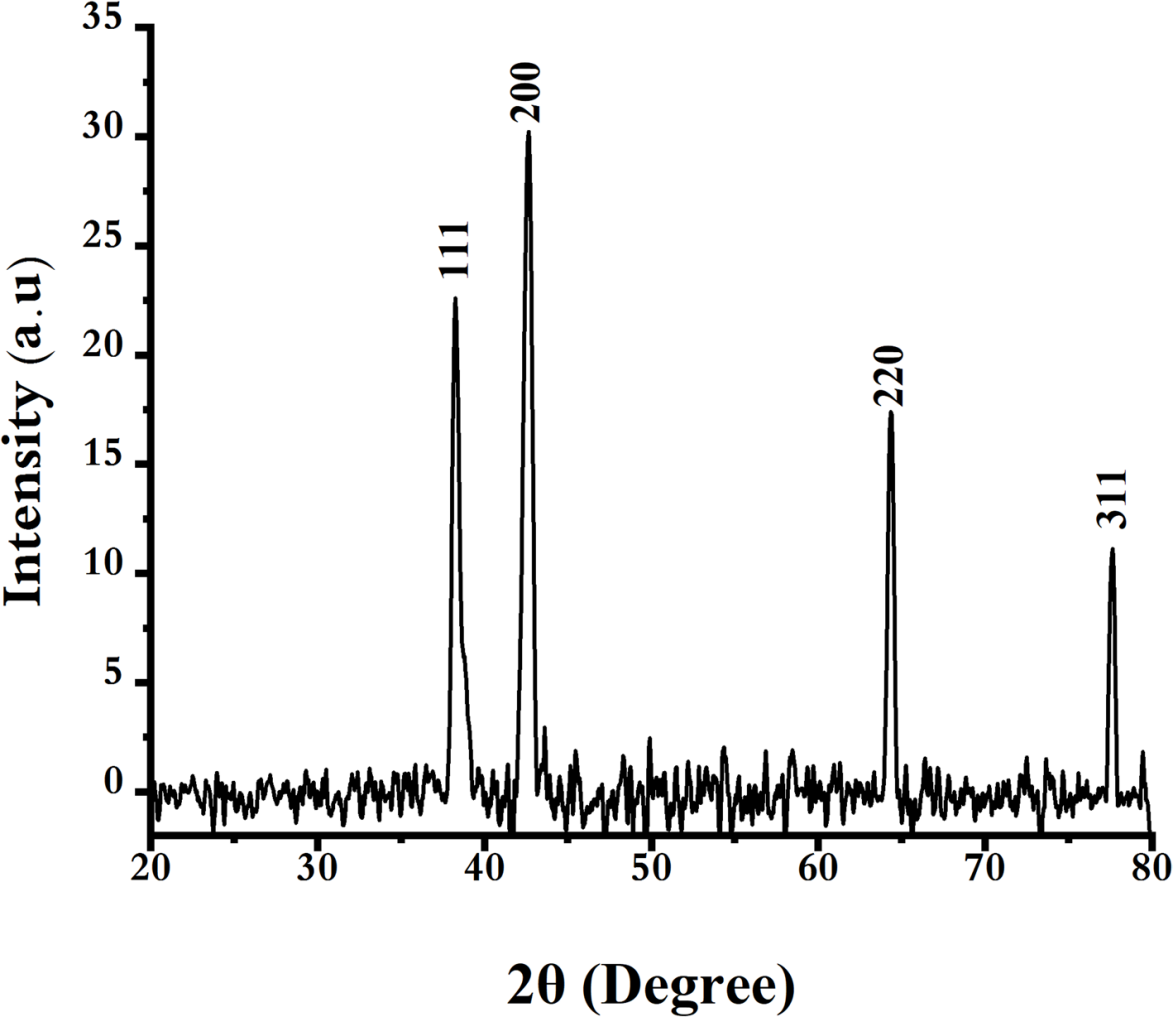
XRD analysis of biosynthesized SLC- AgNPs

XRD is a key technique for characterizing the crystal structure and properties of nanomaterials. XRD analysis was conducted to investigate the crystal structure, phase, and purity of the biosynthesized SLC-AgNPs, as shown in Fig. 7. The XRD data showed typical reflections of the face-centered cubic (fcc) structure of AgNPs (JCPDS, Card No. 04–0783) at 2θ values of 38.2°, 42.6°, 64.4°, and 77.6°.Previously reported XRD analyses demonstrate distinct diffraction peaks characteristic of a crystalline structure in the FFC sample, confirming its well-defined phase composition [54]; [55]; [56]. This pattern verified the crystalline nature of the SLC-AgNPs produced by reducing Ag^+^ with SLC extract [57]; [58]. In addition, the crystallite size was measured using Scherrer‘s equation (Eq. 5):

5 $${\mathrm{D}}= \, \frac{K\lambda }{\beta cos\theta }$$

where K is the Scherrer‘s standard and its number is 0.9. λ is the X-ray source, which is equal to (0.154 nm). The radian degree value of the FWHM of the analyzed peaks is represented by the β symbol. Additionally, θ represents the orientation of the peak in radians. Depending on the reflection, the size of the crystallites, as estimated by analyzing the pattern‘s XRD peaks, was 15 nm.

The lattice parameter of the biosynthesized SLC-AgNPs was estimated using X-ray diffraction (XRD) analysis and Bragg‘s Law. The detected XRD peaks at 2θ values 38.2°, 42.6°, 64.4°, and 77.6°, correspond to the (111), (200), (220), and (311) crystallographic planes of the face-centered cubic (fcc) structure of AgNPs (JCPDS Card No. 04–0783), confirming the FCC structure of the silver NPs. Using the interplanar spacing (d) and the FCC lattice equation, the lattice parameters for these planes were calculated as 4.07 Å, 4.25 Å, 4.09 Å, and 4.07 Å, respectively. The average lattice parameter was found to be 4.08 Å indicating, which is an indication of their high crystallinity and a well-defined structure.

**Fig. 8:**
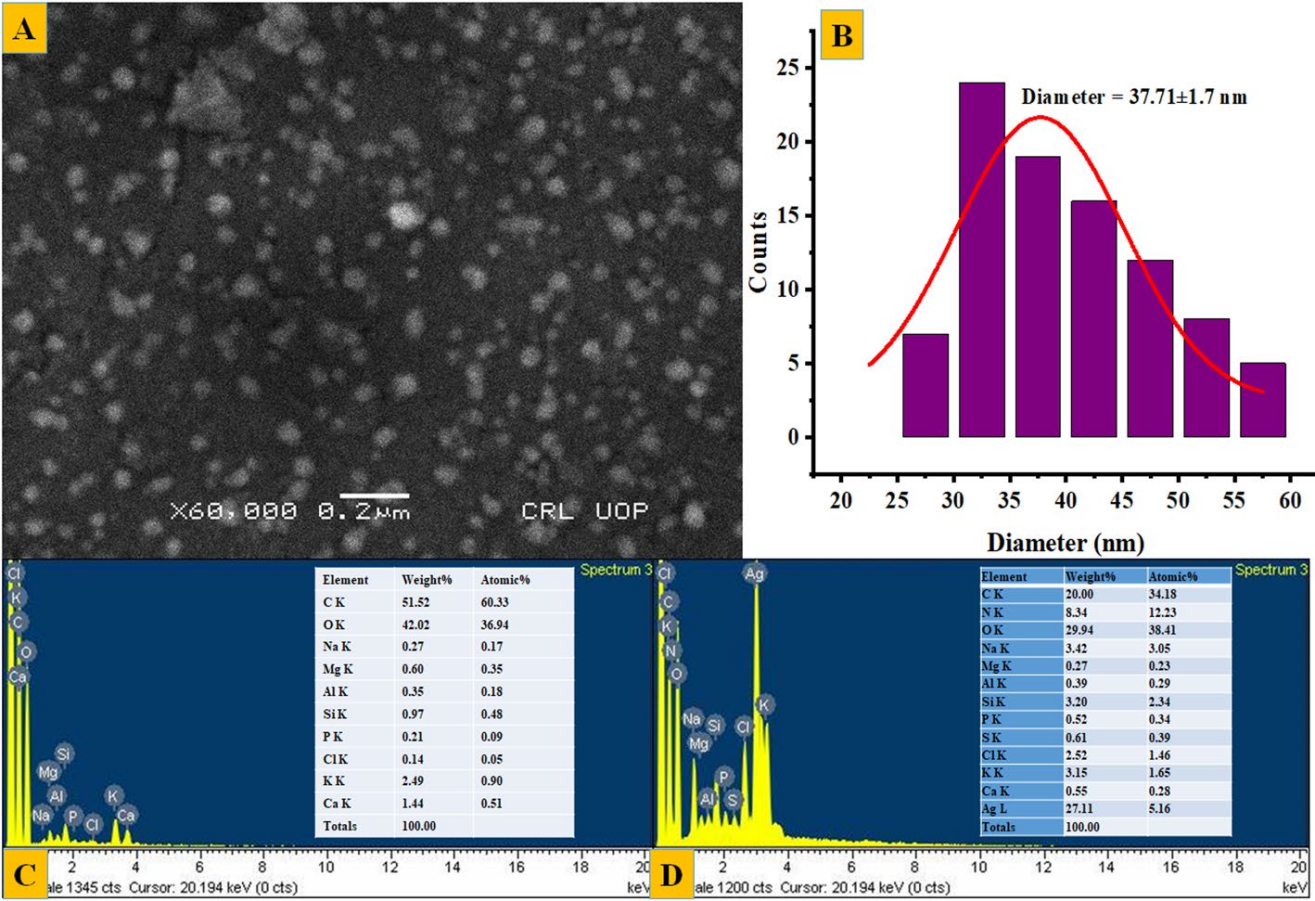
**A** SEM analysis of SLC-AgNPs, **B** Histogram of size distribution accompanied by the lognormal curve; the average size along with the standard deviation is provided, **C** EDX pattern of SLC extract and **D** EDX pattern of SLC-AgNPs

The surface morphology of the biosynthesized SLC-AgNPs was analyzed by SEM, as shown in Fig. 8A. The spherical particles, formed without significant aggregation with an average size of 37.7 ± 1.7 nm, as shown in Fig. 8B. Furthermore, to determine the elemental composition, EDX spectra were obtained for both the SLC extract and the SLC-AgNPs samples. The EDX spectra revealed strong signals for Ag in the SLC-AgNPs sample, confirming the presence of AgNPs, as shown in Fig. 8C. The elemental composition of the SLC-AgNPs was calculated, showing 27.11% Ag, 29.94% oxygen by weight, 20.00% carbon, and some other elements, which might be due to the plant extract. The EDX spectra of the plant extract are shown in Fig. 8D. However, the plant extract did not show any Ag signal. The presence of indications for S, K, Ca, Si, Mg, Na, P, N, Cl, and Al indicated that the extract contains phytochemical components. Compared with the uncapped extract, the capped AgNPs showed a decrease in the C and O signals.

**Fig. 9:**
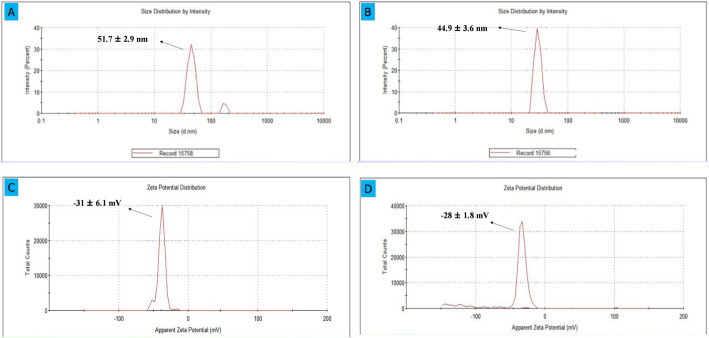
**A** DLS analysis and, **B** DLS analysis showing a decrease in SLC-AgNPs‘ hydrodynamic size after Hg^2^⁺ interaction with rising Hg^2^⁺ concentration, **C** Zeta Potential analysis of SLC-AgNPs, **D** Zeta potential of SLC-AgNPs after 2 months of storage

The hydrodynamic diameter of SLC-AgNPs was estimated using DLS zeta sizer analysis, as shown in Fig. 9A. The measured size of the NPs was 51.7 ± 2.9 nm. There were very few aggregates, and the particles exhibited excellent dispersion in water for this size range. The size determined by DLS analysis was larger than that observed by SEM and UV–vis examination [59]; [60]^.^ This difference may be due to the presence of a solvation shell around the AgNPs which contributes to the bigger size measured by DLS [57]. Furthermore, DLS measurements are based on intensity, while SEM pictures provide the distribution of particle numbers. Zeta potential, a fundamental concept in nanotechnology, governs the interactions and stability of NPs. The stability and aggregation of particles may be inferred from zeta potential (ZP) measurements, which regulate the kind and intensity of their surface charges [57]; [59]. The long-term colloidal stability of SLC-AgNPs was evaluated using zeta potential measurements. As shown in Fig. 9C, the freshly synthesized NPs exhibited a strong negative surface charge, indicating good electrostatic repulsion and dispersion stability. After 2 months of storage at room temperature, the zeta potential remained relatively stable at -28 ± 1.8 mV (Fig. 9D), confirming that the NPs maintained their surface charge and did not undergo significant aggregation or degradation over time. This result demonstrates the effective capping and stabilizing ability of the *S. lycopersicum* extract, supporting the long-term usability of the synthesized AgNPs.

**Fig. 10:**
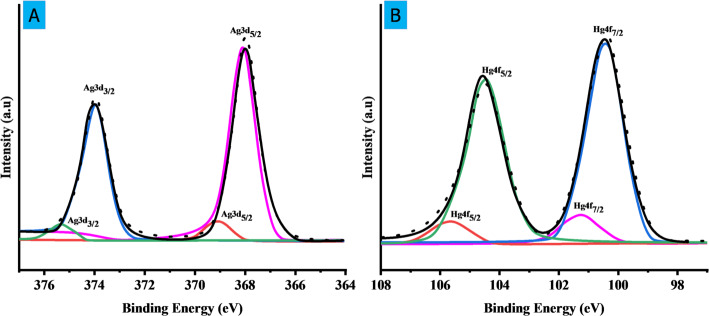
**A** XPS spectrum of Ag 3d region showing peaks for Ag⁰ and Ag⁺, confirming the mixed oxidation states of silver in SLC-AgNPs, **B**. High-resolution XPS spectrum of Hg 4f region showing peaks at ~ 100.6 eV (Hg 4f₇/₂) and ~ 104.6 eV (Hg 4f₅/₂), confirming the reduction of Hg^2^⁺ to elemental mercury (Hg⁰) on the AgNP surface

The XPS analysis of SLC-AgNPs reveals two major peaks at 368.1 eV (Ag 3d₅/₂) and 374.1 eV (Ag 3d₃/₂), which are characteristic of metallic silver (Ag⁰). These peaks confirm the successful reduction of Ag⁺ to Ag⁰ during the green synthesis using *S. lycopersicum* leaf extract as shown in Fig. 10A. In addition to the main Ag⁰ signals, smaller peaks at slightly higher binding energies (~ 369.3 eV and ~ 375.3 eV) are also observed. These are assigned to oxidized silver species (Ag⁺), likely present as Ag₂O or surface-bound Ag⁺ ions.

The presence of both Ag⁰ and Ag⁺ suggests a mixed valence state on the nanoparticle surface, which is common in green-synthesized AgNPs due to the dual role of plant phytochemicals-as reducing agents and stabilizing or capping ligands. Functional groups such as hydroxyl, carbonyl, and carboxylic acid moieties from flavonoids and phenolics may interact with silver ions, leading to partial oxidation and the formation of Ag–O or Ag–C bonds. This partial oxidation enhances surface reactivity while the phytochemical capping layer prevents aggregation and contributes to colloidal stability. Moreover, the coexistence of Ag⁰ and Ag⁺ on the nanoparticle surface renders the system redox-active, which is essential for its function as a selective sensor for Hg^2^⁺. The availability of surface Ag⁰ enables redox exchange with Hg^2^⁺, while the Ag⁺ species represent surface-bound forms stabilized by coordination with plant extracts. This dual chemical environment primes the nanoparticles for efficient electron transfer, which is crucial for sensing applications and photocatalytic activity. Wu et al. reported Ag 3d doublets at ~ 367.5 eV and 373.5 eV corresponding to Ag⁰, along with O 1 s peaks between 530.9 and 533.4 eV, indicating organic capping by Moringa extract [61]. Similarly, Alamar et al. confirmed the co-existence of Ag⁰ and AgO/Ag₂O on AgNP-coated cotton fibers, with supporting C 1 s and O 1 s signals confirming biomolecular interactions [62]. Our XPS data, which show similar Ag⁰/Ag⁺ binding energies along with C and O signals, are consistent with these findings and strongly support the hypothesis that phytochemicals from *S. lycopersicum* effectively cap and stabilize the NPs, resulting in partial surface oxidation and redox reactivity.

### Selective colorimetric sensing of metal ions using SLC-AgNPs

**Fig. 11:**
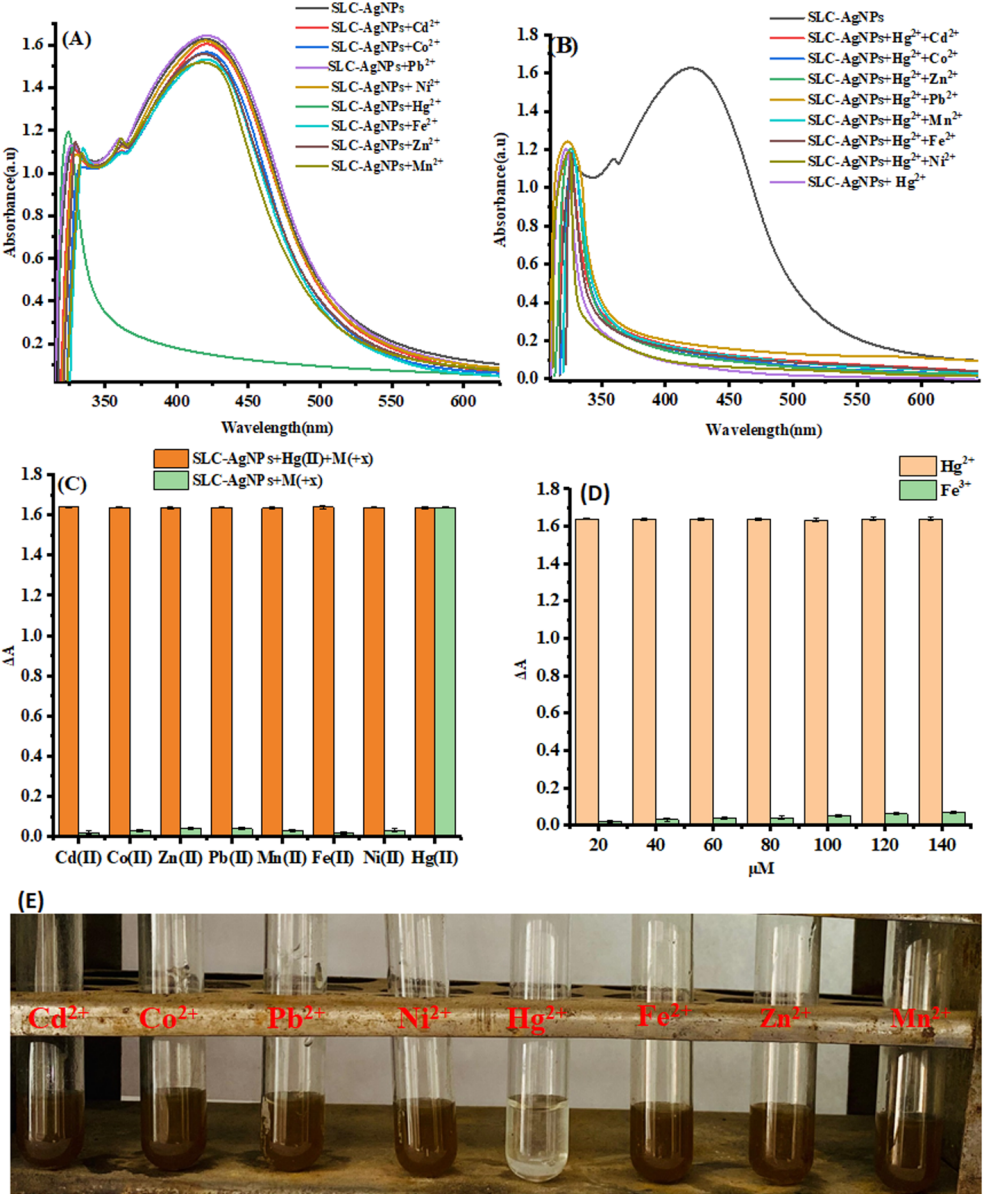
**A** UV–Vis spectra showing selectivity of SLC-AgNPs toward Hg^2^⁺. **B** Spectra of SLC-AgNPs with Hg^2^⁺ and other metal ions. **C** Bar chart illustrating interference effects. **D** Sensitivity plot showing linear ΔA response for Hg^2^⁺ (20–140 nM). **E** Visual color change of SLC-AgNPs with various metal ions confirming selective detection of Hg^2^⁺

The sensing capability of the SLC-AgNPs was evaluated by studying their interactions with Cd^2+^, Co^2+^, Pb^2+^, Ni^2+^, Fe^2+^, Zn^2+^, and Mn^2+^. The UV–vis spectra Fig. 11A were recorded after adding equal amounts of SLC-AgNPs solution to each metal ion solution (1 mM). Figure 11E shows that the mixture’s color changes in the presence of Hg^2+^, and Fig. 11A and B shows that the distinctive LSPR peak of AgNPs was diminished in the UV–vis spectra. There was no discernible change in color or shift in the UV–vis spectra for mixtures of other metal ions with SLC-AgNPs. The ability to selectively detect Hg^2+^ ions in solution was demonstrated by the color change and LSPR peak shift observed with these ions. The fundamental process behind AgNPs’ preferential interaction with Hg^2+^ is shown in Fig. 11B, which displays the change in absorbance (ΔA) of the LSPR peak after interaction with different metal ions compared to the control AgNPs solution. It is evident that, following contact with all metal ions under investigation, AgNPs exhibited only minimal changes in absorbance, except for Hg^2+^, which demonstrated a notable shift. Therefore, these SLC functionalized AgNPs demonstrated good selectivity for Hg^2+^ ion detection.

### Selective detection of Hg^+2^ ions using colorimetric assay

**Fig. 12:**
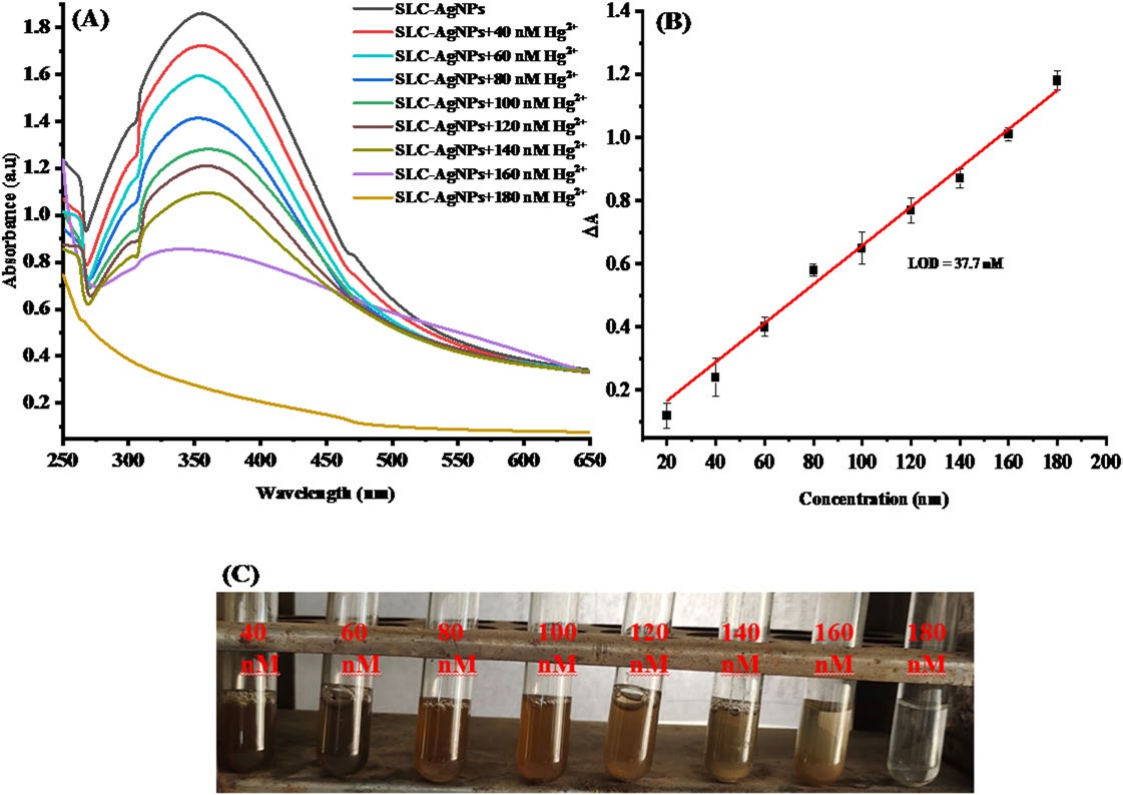
**A** UV–vis spectra of the SLC-AgNPs solution following the introduction of varying concentrations of Hg^2+^, **B** Linear representation of the difference in relative absorbance intensity at peak maxima as a function of Hg^2+^ concentration and **C** Image depicting various solutions of Hg^2+^ at varying concentrations

The World Health Organization (WHO) recommends keeping mercury levels in drinking water below 1 µgL^−1^, and the Environmental Protection Agency ( EPA) has a strict limit of 2 µgL^−1^ [63]. The present methods for determining Hg^2+^ in water samples have several limitations, including susceptibility to interferences and the need for specific reagents. A different approach that is direct, perceptive, and responsive is required to solve these limitations. In this work, this was achieved by using SLC-AgNPs as a quick nanosensor. Various amounts of Hg^2+^ ranging from 40 to 180 nM were added to the SLC-AgNPs solution for quantitative analysis. Notably, that this technique enabled the detection of Hg^2+^ with the unaided eye. The images in Fig. 11C demonstrate how AgNPs visually quench as Hg^2+^ concentration rises. To quantify this color change, we recorded and analyzed the UV–vis spectra of these solutions. Figure 11A shows that as the quantity of Hg^2+^ ions increased, the UV–vis absorbance decreased. The decrease in absorbance with increasing Hg^2^⁺ concentration was found to be statistically significant (p-value (1.99 × 10^–18^), one-way ANOVA), confirming the sensor’s high sensitivity and linearity Table 1S. By plotting a calibration curve of Hg^2^⁺ concentration versus the change in absorbance, the limit of detection (LOD) was determined (Fig. 12B). A strong linearity between 40 and 180 nM was shown by the correlation coefficient (R^2^) of 0.989, as shown in Fig. 12B. The LOD was determined to be 37.7 nM using Equation (Eq. 6).

**Table 1:** Real water sample analysis of the synthesized SLC-AgNPs as nano-probe

	Concentration (nM)	Found Hg^+2^ (nM)	% recovery		Found Hg^+2^ (nM)	% recovery	
River Water	40	32.18	82.95	Tap Water	31.18	83.95	
	60	52.18	83.3		50.18	84.3	
	80	60.58	84.22		63.58	85.22	
	100	75.19	85.18		77.19	86.18	
	120	95.18	86.65		95.18	86.65	
	140	101.59	87.55		100.59	87.11	
	160	121.38	88.73		119.38	88.23	
	180	132.17	89.43		130.17	89.02	
	Average	86.00			Average	86.33	
	Standard deviation	2.46			Standard deviation	1.79	
	SE of intercept		0.345			SE of intercept	0.257
	SD of intercept	0.977			SD of intercept	0.727	
	%RSD	2.86			%RSD	2.08	
	LOD	45.9			LOD	46.1	
	LOQ	139.22			LOQ	139.54

6 $$LOD=\frac{3\left(\text{Standard deviation of intercept}\right)}{\text{Slope of the calibration curve}}$$

### EDX mapping

**Fig. 13:**
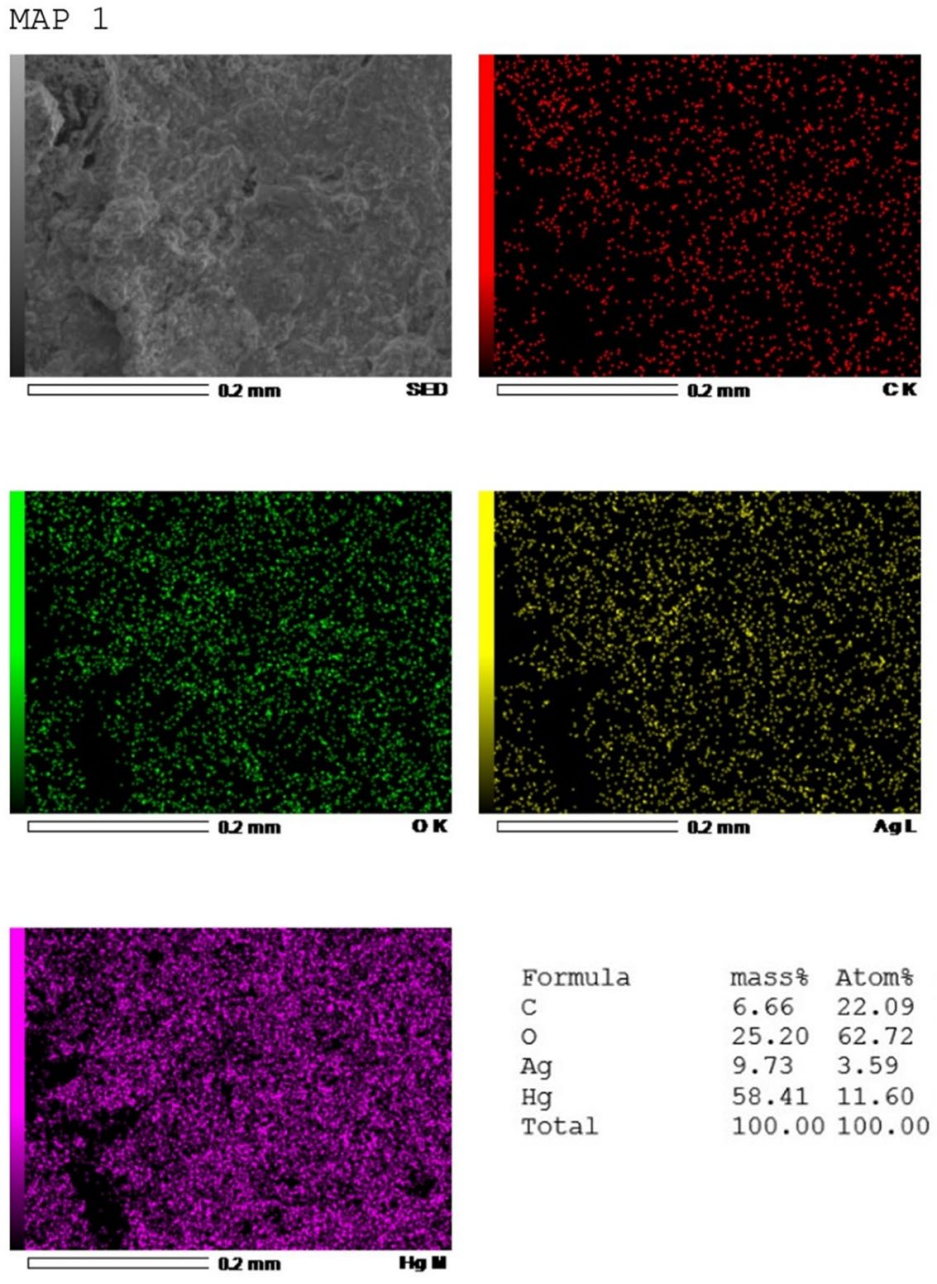
Energy Dispersive X-ray Spectroscopy elemental mapping and composition analysis of SLC-AgNPs after interaction with Hg^2^⁺

The EDX mapping analysis provides essential information on the distribution of Hg(II) and silver atoms after the oxidation of AgNPs with varying concentrations of Hg(II). The redox reaction occurs as Hg^2^⁺ is applied, with the mechanism involving the oxidation of metallic silver (Ag⁰) to Ag⁺ while Hg^2^⁺ is reduced to elemental mercury (Hg⁰), which ultimately deposits on the surface of the metallic nanoparticles. This alteration is apparent in EDX mapping, where increasing Hg(II) concentrations leads to a gradual decrease in the Ag signal and an increased distribution of Hg, confirming the oxidizing behavior of AgNPs and the plating of Hg. The elemental maps further support this evolution showing a low intensity Ag signal (yellow) due to Ag oxidation and a rising Hg M signal (magenta), indicating Hg accumulation as shown in Fig. 13. The EDX spectrum clearly shows an increased amount of Hg, demonstrating the successful adsorption and reduction of Hg(II). These findings support the idea of AgNPs acting as selective mercury (II) probes.

### Size Variation of AgNPs after Hg^2^⁺ interaction

The hydrodynamic size distribution of SLC-AgNPs before and after sensing Hg^2^⁺ was evaluated using Dynamic Light Scattering (DLS). As shown in Fig. 9A, the average particle size of the NPs before sensing was 51.7 ± 2.9 nm. After exposure to Hg^2^⁺, the average size decreased noticeably to 44.9 ± 3.6 nm (Fig. 9B). This reduction is attributed to the redox interaction between Hg^2^⁺ and the metallic Ag⁰ on the nanoparticle surface. In this process, Ag⁰ is oxidized to Ag⁺, while Hg^2^⁺ is reduced to Hg⁰. The reaction may lead to partial removal or rearrangement of the outer silver layer and a more compact surface structure, potentially due to the formation of a thin Ag–Hg amalgam shell. This change results in a decrease in the overall hydrodynamic radius, as detected by DLS. This phenomenon is well-documented—for example, DLS analysis of AgNPs exposed to low concentrations of Hg^2^⁺ (30 µM) also showed a decrease in size, attributed to etching and alloy formation from Hg reduction [20]; [64].The observed size contraction supports the redox-based sensing mechanism and confirms chemical interaction at the nanoparticle surface.

### Interference study

Additionally, a study was carried out to examine the potential interference caused by different metal ions during the sensing of Hg^2^⁺ ions. For this experiment, we mixed SLC-AgNPs (1 mL) with Hg^2+^ (150 nM, 1 mL) and measured their UV–vis spectra in the presence of Cd^2^⁺, Co^2^⁺, Pb^2^⁺, Ni^2^⁺, Fe^2^⁺, Zn^2^⁺, and Mn^2^⁺ salts (1 mM, 1 mL), as shown in Fig. 11B. Even at increasing concentrations, the absorbance of the spectra declined similarly regardless of the salt solution. The absorbance change of SLC-AgNPs is shown by the green bars, using a 1 mM solution of Cd^2^⁺, Co^2^⁺, Pb^2^⁺, Ni^2^⁺, Fe^2^⁺, Zn^2^⁺, and Mn^2^⁺. The yellow bars illustrate the shift in absorbance of SLC-AgNPs + Hg^2+^ in response to a 1 mM solution of these metal ions [65]. Even at higher concentrations of different salt solutions, the absorbance of the UV–vis spectra shifted in a manner almost identical to that seen with Hg^2+^ ions alone as shown in Fig. 11C. This indicated that, SLC-AgNPs have a good sensitivity and selectivity for Hg^2^⁺ detection. To further evaluate sensor selectivity, we extended the interference study to include ferric ions (Fe^3^⁺) as shown in Fig. 11D. The results showed that Fe^3^⁺ did not induce any significant change in the LSPR peak or colorimetric response, confirming that the sensing mechanism remains highly selective for Hg^2^⁺. Even at higher concentrations of Fe^3+^, the absorbance shift was negligible compared to the response seen with Hg^2^⁺ ions alone. ^2^⁺. This confirms the high selectivity of our sensor for Hg^2^⁺ over trivalent metal ions. The specificity of the sensor toward Hg^2^⁺ over trivalent ions like Fe^3+^ arises from its higher redox potential, enabling oxidation of Ag^0^ and LSPR changes, along with selective surface interactions facilitated by phytochemicals (e.g., flavonoids, phenolics) from S. lycopersicum extract that preferentially bind Hg^2+^ [66]; [20]. Furthermore, to verify the stability and selectivity of the SLC-AgNPs under realistic conditions, possible interference from common anions (Cl^−^, NO₃^−^, SO₄^2−^, and PO₄^3−^) typically present in natural water was also examined (Supplementary Fig. 1S A-C). The results revealed no significant change in the LSPR response toward Hg^2^⁺, confirming that the SLC-AgNPs sensor is highly selective and unaffected by coexisting anionic species. These results further validate the strong selectivity of SLC-AgNPs for Hg^2^⁺ detection in complex aqueous environments.

### The proposed mechanism of heavy metal sensing

**Fig. 14:**
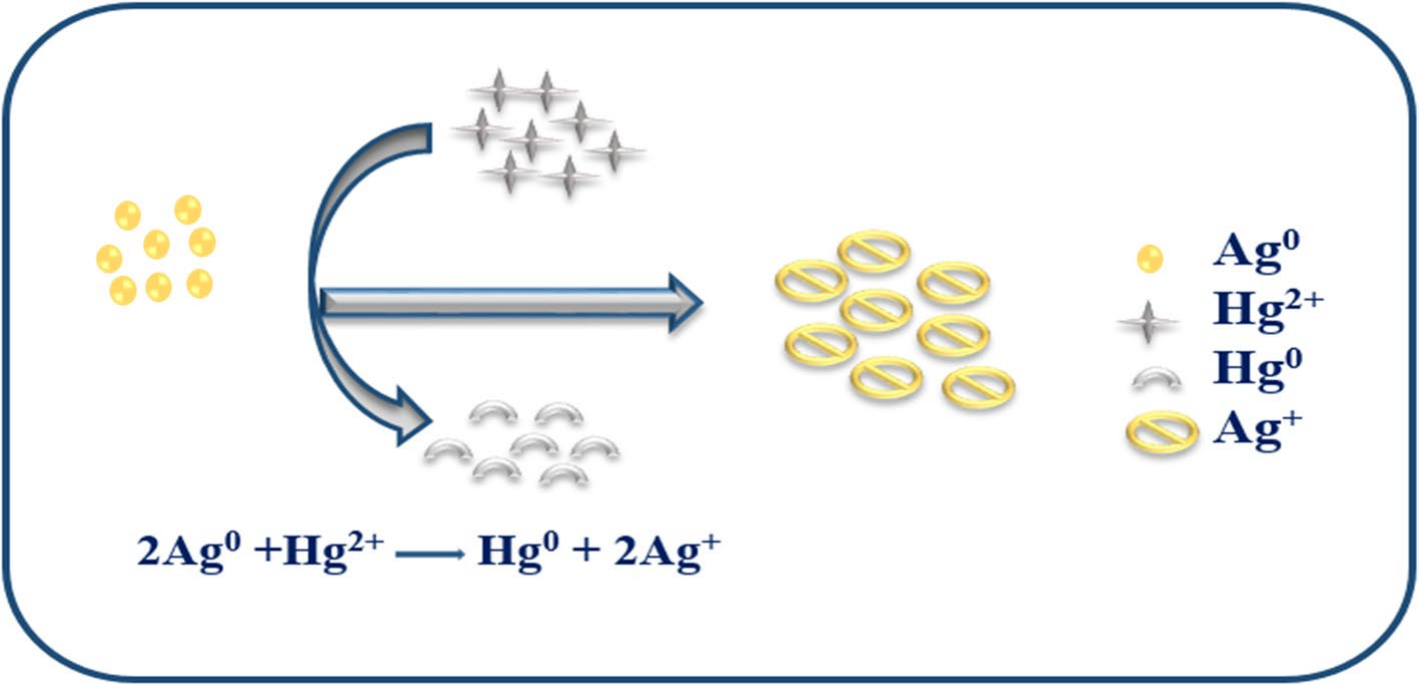
Schematic diagram of redox reaction mechanism

AgNPs can be used in four different methods to detect Hg^2+^. Ag–Hg alloy formation, complexation, aggregation, and redox reaction [67]. In the redox reaction mechanism, Hg^2+^ interacts with Ag atoms, immediately oxidizing Ag^0^ to Ag^+^ and reducing Hg^2+^ to Hg^0^, as shown in Fig. 14. The redshift and expansion of the LSPR band are typical of AgNP redox reactions with metal ions. However, a blue shift is caused by the redox reaction mechanism, even though both processes reduce the LSPR intensity [68]. Metal-ion interaction is often regulated by the capping agent of the NPs. Instead of the conventional aggregation/coalescence pathway, SLC-AgNPs sense Hg^2+^ through redox reactions between Hg^2+^ and Ag^0^. A noticeable decrease in hydrodynamic size from 51.7 ± 2.9 nm to 44.9 ± 3.6 nm after Hg^2^⁺ exposure indicates redox-induced surface modification of AgNPs. This suggests oxidation of surface Ag⁰ to Ag⁺ and concurrent reduction of Hg^2^⁺ to Hg⁰, possibly forming an Ag–Hg interface. Simulated XPS results further support this mechanism, showing shifts in Ag 3d peaks and the appearance of Hg 4f signals, confirming the chemical transformation. The high-resolution XPS spectrum of the Hg 4f region confirms the redox interaction between Hg^2^⁺ and SLC-AgNPs. Two prominent peaks were observed at ~ 100.6 eV and ~ 104.6 eV, corresponding to Hg 4f₇/₂ and Hg 4f₅/₂, respectively As shown in Fig. 10B. These binding energies are characteristic of elemental mercury (Hg⁰), indicating that Hg^2^⁺ was reduced on the AgNP surface. Deconvolution of the spectrum further revealed minor contributions from Hg^2^⁺ species, suggesting incomplete reduction or the presence of surface-bound oxidized mercury. The detection of Hg⁰ strongly supports the proposed redox mechanism, wherein Hg^2^⁺ oxidizes Ag⁰ to Ag⁺ and itself gets reduced and deposited on the nanoparticle surface, possibly forming an Ag-Hg amalgam. The LSPR shifts to becomes blue as the concentration increases. This behavior is action was consistent with another approach for detecting Hg^2+^ using AgNPs [20]. With the reduction of Hg^2+^ to Hg^0^ and the oxidation of Ag^0^ to Ag^+^, the color of the solution changes from yellowish brown to colorless, as seen in Fig. 11E, when Ag oxidizes to Ag^+^ in SLC-AgNPs Ag^+^/Ag and Hg^2+^/Hg have equilibrium redox potentials of + 0.80 and + 0.85 V, respectively [69]. The redox potentials demonstrate that Ag can be oxidized to Ag^+^ and then reduced to metallic Hg atoms by Hg^2+^, which has a greater reduction potential than Ag^+^. Contrarily, transition and alkaline earth metals with more negative reduction potentials than Ag (+ 0.80 V) -such as Cd^2+^, Co^2+^, Pb^2+^, Ni^2+^, Fe^2+^, Zn^2+^, and Mn^2+^- made it more difficult for Ag atoms in SLC- AgNPs to oxidize to Ag^+^ [70]. Thus, the hue of the solution was not changed by other metal ions. This behavior makes the approach particularly interesting for selective Hg^2+^ analysis. In terms of LOD values and linear dynamic range, the present nano-sensor performs better than several sensors documented in the literature. It was also key to investigate the potential of this sensor for detecting Hg^2+^.The AgNP-Hg^2+^ interaction was suggested as the cause of the observed changes in the LSPR of AgNPs. The R^2^ value for the linear regression was 0.989, and the LOD was found to be 37.7 nM as shown in Fig. 12B. Together, these findings strongly validate the proposed redox-based sensing mechanism.

### Real sample analysis

Additional consideration was given to the possibility of detecting Hg^2+^ in river and tap water samples using the synthesized nano-probe. The water sample used in the study was collected from the Jhelum River near Sargodha, Pakistan, while another sample was drawn from the tap at the University of Sargodha‘s Chemistry Laboratory. The water samples were treated with varying concentrations of Hg^2+^. Recovery values and RSD for the real water samples are shown in Table 1. A recovery rate of more than 86% and RSD values of 2.86 and 2.08 were observed in the real water samples. In light of these results, the colorimetric nano-probe may be a reliable and efficient method for identifying Hg^2+^ in real water samples.

#### Comparison with previously reported Hg^2^⁺ detection methods

The sensing performance of the SLC-AgNPs developed in this study was compared with previously reported AgNP-based colorimetric sensors for Hg^2^⁺ detection (Table 2). Several materials, including mPEGylated luteolin-AgNPs, gelatin/Tween-20 stabilized AgNPs, and plant-derived systems such as Kokum fruit or *S. mammosum* extract, have demonstrated good sensitivity with linear ranges from 0.5 to 130 µM and limits of detection (LODs) between 0.0028 and 0.97 µM. In contrast, our SLC-AgNPs achieved a notably lower detection limit of 37.7 nM (0.0377 µM) with a narrow and highly sensitive linear range of 40–180 nM, indicating superior detection capability in the low-nanomolar range. Additionally, our approach offers a rapid, eco-friendly synthesis process (completed in 3 min under sunlight), without the need for stabilizers or complex instrumentation. The dual functionality of these nanoparticles as both selective Hg^2^⁺ sensors and efficient photocatalysts, further enhances their practical relevance for environmental applications.

**Table 2:** Comparison of AgNP-based sensors for Hg^2^⁺ detection

Sr No.	Material/Plant Extract	Linear Range	LOD	References
1	mPEGylated luteolin AgNPs	5–130 µM	0.97 µM	[71]
2	Green AgNPs from Kokum fruit extract	0.01–10 µM	6.2 ppb (~ 0.0062 µM)	[72]
3	Gelatin & Tween 20 stabilized AgNPs	0.5–100 µM	0.35 µM	[73]
4	Glucose capped AgNPs (green method)	1–70 µM	2.8 ppb (~ 0.0028 µM)	[74]
5	SLC-AgNPs	40–180 nM	37.7 nM	This work

#### Photocatalytic activity evaluation

**Fig. 15:**
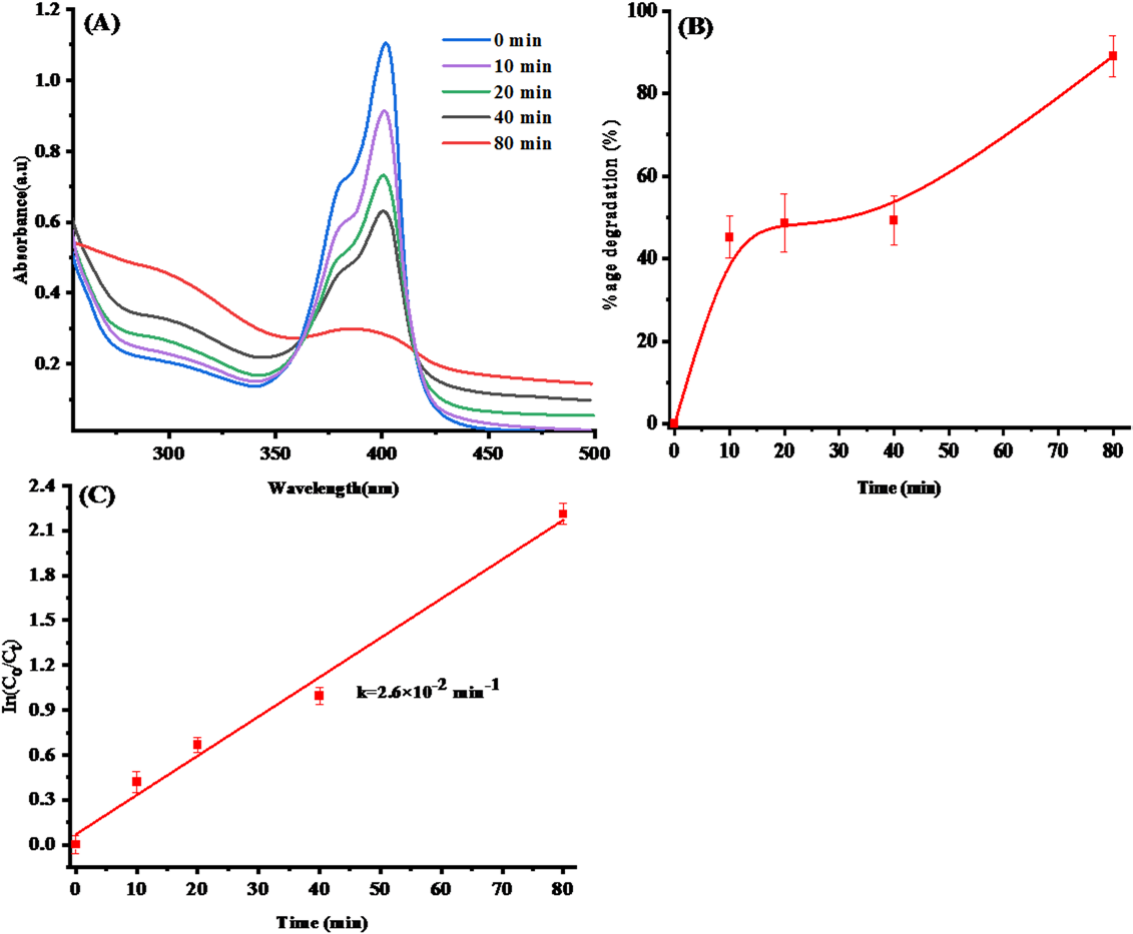
**A** MB dye‘s absorption spectra at regular intervals, **B** the proportion of MB dye % degradation over time, **C** Kinetics of MB dye

Using methylene blue (MB) as a model textile dye, the photocatalytic efficacy of SLC-AgNPs was evaluated. The dye‘s absorption spectra are shown in Fig. 15A at regular time intervals [75]. The following equation was used to calculate degradation efficiency as a percentage (Eq. 7).

7 $${\% dye degradation }= \, \frac{{A}_{0}-{A}_{f}}{{A}_{0}}\times 100$$

Figure 15B shows the percentage degradation of MB dye over time. The plot clearly shows that the SLC-AgNPs exhibited strong catalytic activity, as evidenced by the 83.3% deterioration of the MB solution deterioration in 80 min. The photocatalyst under study outperforms many previously reported materials, demonstrating an exceptional degradation percentage in a very short period. In a previous study, AgNPs treated with *Astragalus flavesces* degraded MB dye by 69% in 28 h when exposed to sunlight [76].

#### The kinetics of MB degradation and thermodynamic parameters

ln(C_0_/C_t_) was plotted against irradiation time to investigate the kinetics of the photocatalytic process. The results are shown in Fig. 15C. The resulting linear relationship is described by q. (8):

8 $$ln\left(\frac{{C}_{0}}{{C}_{t}}\right)=kt$$

C_t_ represents the concentration at time interval t, whereas C_0_ represents the concentration of the reaction mixture at zero reaction time. The rate constant, k, was determined from the slope of the linear fit. The photodegradation process followed pseudo-first-order reaction kinetics, as the MB dye plot was reasonably linear, with an R^2^ value of 0.987.

The degradation of MB dye was examined after exposure to temperatures of 30, 40, and 50 °C for 80 min. We observed the absorbance of MB at 665 nm over time to assess its degradation rate. The rate constant (k) was determined using a pseudo-first order kinetic model. The rate constant values were calculated using the following equation (Eq. 9):

9 $$ \ln \left( {{{A_{0} } \mathord{\left/ {\vphantom {{A_{0} } {A_{t} }}} \right. \kern-0pt} {A_{t} }}} \right) = kt $$

**Fig. 16:**
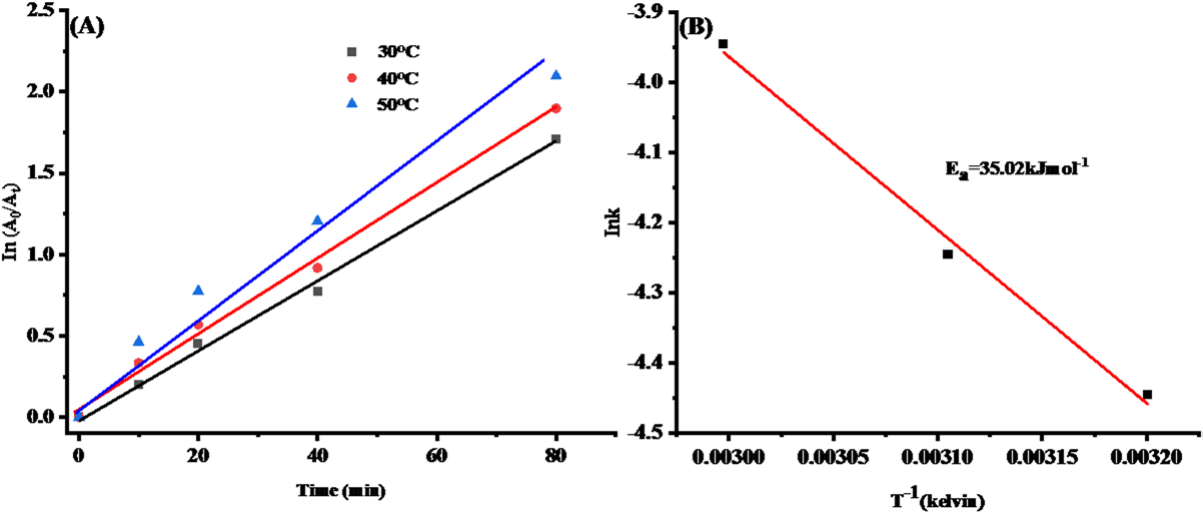
**A** Fitting a first-order kinetics model to the experimental degradation data of MB dye across various temperatures, **B** Graph of ln(k/T) against 1/T

The rate constant for the degradation reaction is represented by k, while the absorbance of MB dye at the initial time and at time t is denoted as A_0_ and A_t_, respectively. The plots of ln (A_0_/A_t_) versus reaction time for MB degradation at different temperatures are presented in Fig. 16A. The slope of the fitted linear lines is utilized to ascertain the rate constants (k). The k values for the MB dye degrading reaction at different temperatures are presented in Table 3. The linear fitting of the data points demonstrates that the degradation of MB dye follows pseudo-first-order kinetics. Furthermore, the linear fit, with R^2^ values close to unity (0.995, 0.998, and 0.994 at 30, 40, and 50 °C, respectively), supports the conclusion that pseudo-first-order kinetics govern the degradation of MB dye. The variation in degradation rates across different temperatures was statistically significant (F = 108.00, *p* = 1.97 × 10^–5^), one-way ANOVA), confirming the influence of thermal activation on the photocatalytic reaction kinetics Table 2S.

**Table 3:** Thermodynamic parameters of the biosynthesized SlC-AgNPs

Temperature (°C)	*k* (min^−1^)	E_a_ (kJ mol^−1^)	ΔH (kJ mol^−1^)	ΔS (kJ mol^−1^ K^−1^)	ΔG (kJ mol^−1^)
30	0.02	35.02	32.42	−0.17	84.8
40	0.03				86.5
50	0.04				88.2

The thermodynamic triplet (ΔS, ΔH, and ΔG) and activation energy (E_a_) were calculated by performing MB dye degradation at three distinct temperatures (30, 40, and 50 °C). E_a_ was determined using the Arrhenius equation (Eq. 10)

10 $$\mathit{ln}k=-\frac{{E}_{a}}{RT}+\mathit{ln}{A}_{t}$$

Here, k represents the rate constant for the degradation reaction, R denotes the gas constant with a value of 8.314 J mol^−1^ K^−1^, and A signifies the Arrhenius factor. The graph of ln k against 1/T is presented in the inset of Fig. 16B. The slope from the linear fit of the ln k versus 1/T plot was utilized to ascertain E_a_. The determined value of E_a_ is 35.02 kJ mol^−1^.

Equation Eyring-Polanyi was used to calculate the values of ΔS and ΔH of activation (Eq. 11).

11 $$ \ln \left( {{\raise0.7ex\hbox{$k$} \!\mathord{\left/ {\vphantom {k T}}\right.\kern-\nulldelimiterspace} \!\lower0.7ex\hbox{$T$}}} \right) = - {\raise0.7ex\hbox{${\Delta H}$} \!\mathord{\left/ {\vphantom {{\Delta H} {RT}}}\right.\kern-\nulldelimiterspace} \!\lower0.7ex\hbox{${RT}$}} + {\raise0.7ex\hbox{${\Delta S}$} \!\mathord{\left/ {\vphantom {{\Delta S} R}}\right.\kern-\nulldelimiterspace} \!\lower0.7ex\hbox{$R$}} + \ln \left( {{\raise0.7ex\hbox{${k_{B} }$} \!\mathord{\left/ {\vphantom {{k_{B} } h}}\right.\kern-\nulldelimiterspace} \!\lower0.7ex\hbox{$h$}}} \right) $$

Here, k is the rate constant and kB is the Boltzmann constant. As seen in Fig. 16B, plotting ln(k) versus 1/T produces a straight line. The slope and intercept of the plot provide the values of ΔH and ΔS. The activation free energy, ΔG, was then determined using the following equation (Eq. 12).

12 $$\Delta G=\Delta H-T\Delta S$$

Table 3 displays the calculated values of ΔS, ΔH, and ΔG. The data show that lower activation energies are correlated with higher rate constants, and reactions with lower activation energies proceed at greater rates, suggesting that these reactions surpass the activation energy barrier. In this context, ΔH is + 32.42 kJ mol^−1^ and ΔS is  −0.17 kJ mol^−1^ K^−1^. The degradation of MB dye is an endothermic process requiring energy input, as indicated by the positive ΔH value. The negative ΔS value indicates a decrease in disorder as the process, reflecting the progresses and shows energy required for the reaction to proceed. The reaction is not spontaneous, as the positive ΔG value exceeds 84 kJ mol⁻^1^. The thermodynamic values collectively confirm that SLC-AgNPs mediated MB degradation is an endothermic, surface-mediated, and energy-dependent process that requires efficient light activation. These findings highlight the relevance of SLC-AgNPs as an active photocatalyst with high potential for application in solar powered wastewater treatment operations.

#### Reusability test of the photo-catalyst

**Fig. 17:**
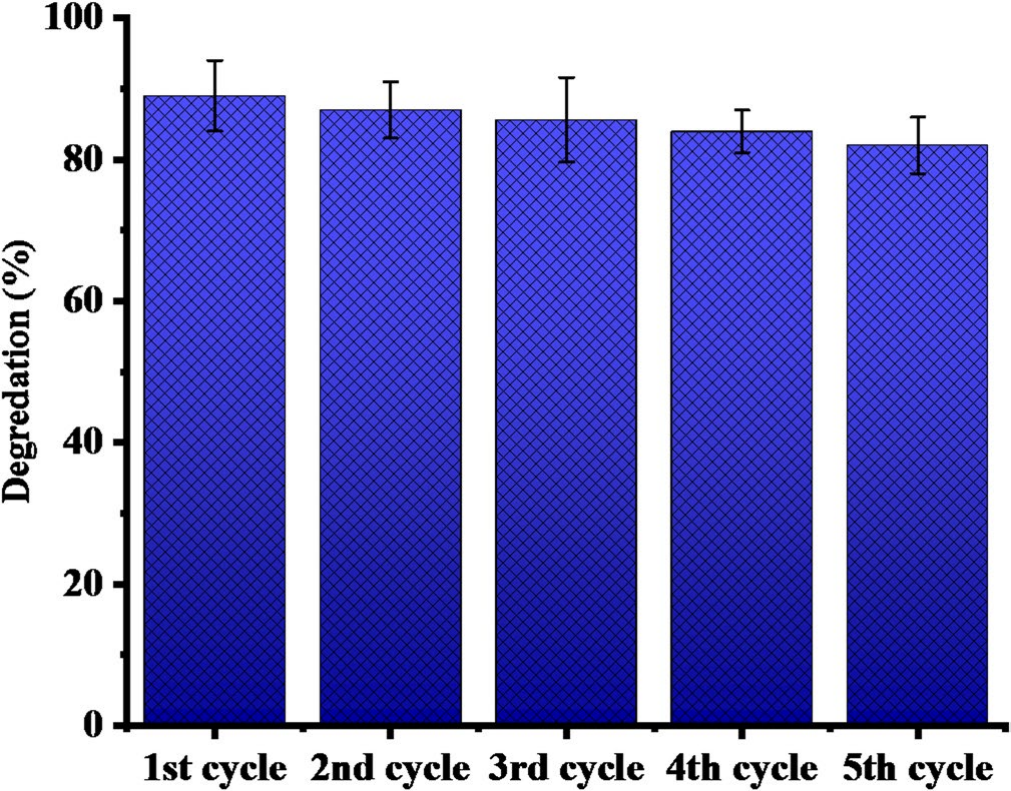
Regeneration cycles of the biosynthesized SLC-AgNPs as catalyst

The potential for reusing SLC-AgNPs as a photocatalyst over multiple cycles was investigated. SLC-AgNPs were employed to degrade MB. After the first cycle of MB dye degradation, the photocatalyst was obtained by centrifuging the mixture in a falcon tube for 25 min at 6000 rpm. The liquid portion was eliminated. The SLC-AgNPs were rinsed three times with deionized water and then dried they were subjected to drying in an oven at 60 °C for 12 h. The dried photo-catalyst was then used for subsequent MB dye photodegradation. The dye degradation and regeneration process was repeated five times. Following each cycle, the percentage of dye degradation was calculated, as Fig. 17 illustrates. After five regeneration cycles, there was a minor (10%) decrease in MB degradation efficiency. These results show that SLC-AgNPs can be repurposed for wastewater treatment and hazardous dye degradation with great efficacy and efficiency, without significant loss of catalytic activity. In a previous study, Panneerselvi et al., (2022) observed a photocatalytic activity of biosynthesized AgNPs for to five cycles, with MB dye degradation following a decreasing from 93% (first cycle) to 89% (fifth cycle) [77]. In another study, Vinay et al., (2019) reported ~ 77% MB dye degradation using biosynthesized AgNPs over six cycles [78].

#### Proposed mechanism of dye degradation

In the photochemical degradation of MB dye by SLC-AgNPs, the photocatalyst was activated by absorbing energy from solar radiation. The energy must be equal or greater than the band gap energy of the photocatalyst. Using the electronegativity approach, the theoretical band edge potentials of AgNPs were calculated as -0.24 eV for the conduction band (CB) and 2.28 eV for the valence band (VB). Since the CB potential is more negative than the O₂/O₂⁻ redox potential (-0.33 eV), AgNPs can effectively produce superoxide radicals (O₂⁻·) which are important for dye degradation. However, the VB potential is less positive than the OH⁻/·OH redox potential (2.8 eV), making OH radical formation unfavorable. This implies that O₂⁻·is the major reactive oxygen species (ROS) responsible for the degradation of methylene blue and the photocatalytic activity of SLC-AgNPs is remarkable. A hole is formed in the valence band and the degradation process begins when electrons in the valence band are excited to the conduction band, as shown in (Eq. 13).

13 $$SLC-AgNPs+h\upsilon \left(UV\right)\to \mathrm{SLC}-AgNPs [{h}^{+}(VB)+{e}^{-}(CB)]$$

An electron oxidizing oxygen in the conduction band produced the superoxide radical, as shown in (Eq. 14)

14 $$ O_{2} + SLC - AgNPs\,[e^{ - } \left( {CB} \right)] \to O_{2}^{ - \cdot } $$

Hydroxyl radicals were created when water molecules encountered oxidation in the valence band hole. As illustrated in the equation, on the other hand, by producing OH^•^ radicals, the positively charged holes were able to neutralize hydroxyl groups (Eq. 15).

15 $${H}_{2}{O}_{(ads)}+\mathrm{SLC}-AgNPs{\hspace{0.17em}}{[h}^{+}(VB)]\to {H{O}_{(ads)}}^{-}+{H}^{+}$$

Equation (Eq. 16) stated that the superoxide radical was neutralized by the protons produced

16 $$ O_{2}^{ - \cdot } \,_{{\left( {ads} \right)}} + H^{ + } \rightleftarrows HOO^{ - } \,\,_{{\left( {ads} \right)}} $$

The HOO − radical, which was generated as indicated above, underwent a reaction to generate hydrogen peroxide and oxygen gas, as shown by the equation (Eq. 17)

17 $$2HO{O}^{-}{{\hspace{0.17em}}}_{\left(ads\right)}\to {H}_{2}{O}_{2}+{O}_{2 {{\hspace{0.17em}}}_{\left(ads\right)}}$$

Hydroxyl free radicals were produced when the generated hydrogen peroxide degraded, as indicated by equation (Eq. 18)

18 $$ H_{2} O_{2} \,_{{\left( {ads} \right)}} \to 2HO^{ \cdot } \,\,_{{\left( {ads} \right)}} $$

A reduced product was produced when electrons in the conduction band decreased the MB dye, as shown in equation (Eq. 19)

19 $$MB{\hspace{0.17em}}Dye+\mathrm{SLC}-AgNPs{\hspace{0.17em}[}{e}^{-}(CB)]\to \mathrm{Re}duction{\hspace{0.17em}}products$$

The MB dye oxidized as a result of the holes in the valence band, forming oxidation products as indicated by equation (Eq. 20)

20 $$MB{\hspace{0.17em}}Dye+\mathrm{SLC}-AgNPs{\hspace{0.17em}[}{h}^{+}(VB)]\to Oxidation{\hspace{0.17em}}products$$

In the degradation process, water and carbon dioxide were produced as a result of a series of intermediate reactions initiated by the interaction between the hydroxide free radical and the MB dye, as shown in the equation (Eq. 21)

21 $$MB{\hspace{0.17em}}Dye+2H{O}^{-}\to intermediates\to C{O}_{2}+{H}_{2}O$$

**Fig. 18:**
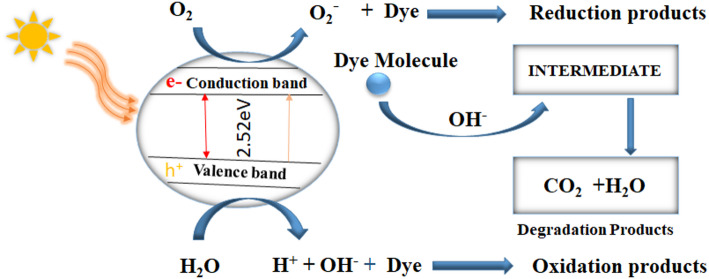
The schematic diagram of cycles illustrates the mechanism of photocatalysis of SLC-AgNPs focused on dye degradation

Figure 18 depicts the schematic of the photocatalytic degradation of MB dyes mediated by SLC-AgNPs.

#### Stability assessment of SLC-AgNPs for dual functional applications

To evaluate the long-term usability of the green-synthesized SLC-AgNPs, stability was monitored over 30 days by evaluating period in terms of both Hg^2^⁺ sensing efficiency and photocatalytic dye degradation. The AgNPs were stored at room temperature in the dark, and measurements were taken at regular intervals. As shown in Table 3S, the absorbance of the SLC-AgNPs at 420 nm gradually declined from 1.20 to 1.10, with the corresponding Hg^2^⁺ sensing response decreasing modestly from 100% to 91.7%. This indicates strong colloidal stability and retention of functional properties. Simultaneously, the photocatalytic activity toward MB showed a progressive decrease in absorbance from 1.00 to 0.65, corresponding to 35% degradation over 30 days. The moderate yet consistent degradation efficiency under ambient conditions without any external irradiation demonstrates the practical usability of the SLC-AgNPs in real-world water remediation scenarios. This functional stability is further supported by the zeta potential measurement (–28 ± 1.8 mV after 60 days), confirming strong electrostatic repulsion and prevention of nanoparticle aggregation over time. These results confirm that the synthesized NPs exhibit stable and reproducible performance for both heavy metal sensing and dye degradation, making them suitable for sustainable environmental applications.

#### Comparison with previous literature

The photocatalytic degradation efficiency of methylene blue (MB) using biosynthesized AgNPs has been widely studied with varying preparation methods and irradiation conditions as shown in Table 4. For instance, AgNPs synthesized from *M. tinctoria* leaf showed 95% degradation under sunlight over 72 h, while AgNPs derived from *A. deliciosa* peel extract achieved 80% degradation in 120 min. Electrochemically prepared AgNPs exhibited 92% efficiency under visible light within 180 min, and honey-mediated AgNPs achieved 92% degradation under sunlight over 72 h. Chemical doping of TiO_2_ with Ag^+^ ions under UV light resulted in 99% degradation within 180 min. AgNPs synthesized from *N. leucophylla* stem extract demonstrated 82.8% degradation under visible light in 180 min. In comparison, our green synthesized AgNPs using *Solanum lycopersicum* leaves extract achieved an 83.4% degradation of MB within only 80 min of sunlight exposure, highlighting a more rapid and efficient photocatalytic process. This demonstrates the potential of our SLC-AgNPs as an eco-friendly and effective photocatalyst for wastewater treatment applications.

**Table 4:** Comparison of photocatalytic degradation efficiency of methylene blue (MB) using various Ag-based nanocatalysts synthesized by different methods or plant extracts under different irradiation conditions

Sr. No	Catalyst	Preparation Method/Plant	Dye	Irradiation	IrradiationTime	DegradationEfficiency/%	References
1	AgNPs	*M. tinctoria* leaf	MB	sunlight	72 h	95	[79]
2	AgNPs	*A. deliciosa peel* extract	MB	Not specified	120 min	80	[80]
3	AgNPs	Electrochemical method	MB	visible light	180 min	92	[81]
4	AgNPs	Honey	MB	sunlight	72 h	92	[82]
5	Ag⁺-doped TiO₂	Chemical doping of TiO₂ with Ag⁺ ions	MB	UV light	180 min	99	[83]
6	AgNPs	*N. leucophylla stem extract*	MB	Visible light	180 min	82.8	[84]
7	AgNPs	*S. lycopersicum* leaves extract	MB	sunlight	80 min	83.4	This work

## Conclusion

This study successfully demonstrates the green synthesis of SLC-AgNPs using *S. lycopersicum* leaves extract, highlighting their dual functionality in selective mercury ion (Hg^2^⁺) detection and photocatalytic degradation of methylene blue (MB) dye. The biosynthesized SLC-AgNPs exhibited a stable crystalline structure with an average particle size of 38 nm, as confirmed by UV–vis, FTIR, XRD, SEM, EDX, and DLS analyses. The interaction of SLC-AgNPs with Hg^2^⁺ was investigated using EDX mapping and DLS analysis revealed a size reduction from 51.7 ± 2.9 nm to 44.9 ± 3.6 nm after Hg^2^⁺ exposure, supporting the redox-based sensing mechanism. The NPs showed excellent selectivity for Hg^2^⁺, with a low detection limit of 37.7 nM and a high recovery rate (> 97%) in real water samples, making them a viable candidate for environmental mercury sensing. Additionally, the SLC-AgNPs demonstrated remarkable photocatalytic efficiency, achieving 83.4% degradation of MB dye within 80 min under sunlight exposure, following pseudo-first-order kinetics with an activation energy of 35.02 kJ/mol. The thermodynamic analysis indicated an endothermic and non-spontaneous nature of the degradation process. Furthermore, recyclability tests confirmed that SLC-AgNPs retained their catalytic efficiency over five successive cycles, with minimal loss of activity. These findings establish SLC-AgNPs as an eco-friendly, cost-effective, and highly efficient nanomaterial for both heavy metal sensing and wastewater treatment. This work underscores the potential of green-synthesized AgNPs as a sustainable alternative to conventional sensing and remediation technologies, paving the way for future applications in environmental monitoring and industrial wastewater management.

## Supplementary Information

Below is the link to the electronic supplementary material.

Supplementary Material 1

## Data Availability

All experimental supporting data and procedures are available within this article.
