# Supplementary material for: Green synthesis of silver nanoparticles using Solanum lycopersicum leaves extract for highly selective detection of mercury ions and photocatalytic degradation of methylene blue
Source: Discov Nano. 2026 Jan 19;21(1):14. doi: 10.1186/s11671-025-04424-2 (PMC12816498; doi:10.1186/s11671-025-04424-2)
Supplement: Supplementary file 1 — Supplementary Material 1 [file 11671_2025_4424_MOESM1_ESM.docx]

Table 1S summarizes the one-way ANOVA performed on absorbance values of SLC-AgNPs at different Hg²⁺ concentrations. A very high F-value (650.36) and extremely low p-value (1.99 × 10⁻¹⁸) indicate that the variation in absorbance is highly significant and attributable to changes in Hg²⁺ concentration rather than random error. This confirms the sensitivity and reliability of the SLC-AgNP-based colorimetric sensor.

Table 1S: Supplementary Table S1: ANOVA Summary for Hg²⁺ Detection

| **Source of Variation** | **Sum of Squares (SS)** | **Degrees of Freedom (df)** | **Mean Square (MS)** | **F-value** | **p-value** |
| --- | --- | --- | --- | --- | --- |
| Between Groups | 0.45525 | 7 | 0.065036 | 650.36 | 1.99 × 10⁻¹⁸ |
| Within Groups | 0.00160 | 16 | 0.000100 | — | — |
| Total | 0.45685 | 23 | — | — | — |

Table 2S reports the mean final absorbance values (At) of methylene blue after 80 minutes of photocatalytic degradation at 30°C, 40°C, and 50°C. The associated ANOVA analysis (F = 108.00, p = 1.97 × 10⁻⁵) demonstrates a statistically significant improvement in degradation efficiency with increasing temperature. This supports the conclusion that photocatalytic activity of SLC-AgNPs is thermally enhanced.

Table S2 – MB Degradation at Different Temperatures. One-way ANOVA: F = 108.00, p = 1.97 × 10⁻⁵

| **Temperature (°C)** | **Mean Absorbance (At)** | **SD** |
| --- | --- | --- |
| 30 | 0.31 | 0.01 |
| 40 | 0.25 | 0.01 |
| 50 | 0.19 | 0.01 |

Table 3S. Stability of SLC-AgNPs over 30 days for Hg²⁺ sensing and methylene blue degradation.

| **Day** | **Absorbance (SLC-AgNPs)** | **Hg²⁺ Response (%)** | **MB Absorbance** | **Dye Degradation (%)** |
| --- | --- | --- | --- | --- |
| 0 | 1.20 | 100.0 | 1.00 | 0 |
| 5 | 1.18 | 98.3 | 0.90 | 10 |
| 10 | 1.16 | 96.7 | 0.85 | 15 |
| 20 | 1.13 | 94.2 | 0.75 | 25 |
| 30 | 1.10 | 91.7 | 0.65 | 35 |

Figure 1S: (A) Selectivity of SLC-AgNPs toward Hg^2+^ over various anions. (B) Interference of other metal ions on the sensing response. (C) ΔA comparison confirming high selectivity for Hg^2+^.
